# Adaptive evolution and metabolic engineering of a cellobiose- and xylose- negative *Corynebacterium glutamicum* that co-utilizes cellobiose and xylose

**DOI:** 10.1186/s12934-016-0420-z

**Published:** 2016-01-22

**Authors:** Jungseok Lee, Jack N. Saddler, Youngsoon Um, Han Min Woo

**Affiliations:** Clean Energy Research Center, Korea Institute of Science and Technology (KIST), Hwarangro 14-gil 5, Seongbuk-gu, Seoul, 02792 Republic of Korea; Department of Wood Science, University of British Columbia, Vancouver, BC V6T 1Z4 Canada; Department of Clean Energy and Chemical Engineering, Korea University of Science and Technology (UST), 217 Gajeong-ro, Yuseong-gu, Daejeon, 34113 Republic of Korea; Green School (Graduate School of Energy and Environment), Korea University, 145 Anam-ro, Seongbuk-gu, Seoul, 02841 Republic of Korea

**Keywords:** *Corynebacterium glutamicum*, Cellobiose and xylose, Cofermentation, Intracellular β-glucosidase, Adaptive evolution

## Abstract

**Background:**

An efficient microbial cell factory requires a microorganism that can utilize a broad range of substrates to economically produce value-added chemicals and fuels. The industrially important bacterium *Corynebacterium glutamicum* has been studied to broaden substrate utilizations for lignocellulose-derived sugars. However, *C. glutamicum* ATCC 13032 is incapable of PTS-dependent utilization of cellobiose because it has missing genes annotated to β-glucosidases (bG) and cellobiose-specific PTS permease.

**Results:**

We have engineered and evolved a cellobiose-negative and xylose-negative *C. glutamicum* that utilizes cellobiose as sole carbon and co-ferments cellobiose and xylose. NGS-genomic and DNA microarray-transcriptomic analysis revealed the multiple genetic mutations for the evolved cellobiose-utilizing strains. As a result, a consortium of mutated transporters and metabolic and auxiliary proteins was responsible for the efficient cellobiose uptake. Evolved and engineered strains expressing an intracellular bG showed a better rate of growth rate on cellobiose as sole carbon source than did other bG-secreting or bG-displaying *C. glutamicum* strains under aerobic culture. Our strain was also capable of co-fermenting cellobiose and xylose without a biphasic growth, although additional pentose transporter expression did not enhance the xylose uptake rate. We subsequently assessed the strains for simultaneous saccharification and fermentation of cellulosic substrates derived from Canadian Ponderosa Pine.

**Conclusions:**

The combinatorial strategies of metabolic engineering and adaptive evolution enabled to construct *C. glutamicum* strains that were able to co-ferment cellobiose and xylose. This work could be useful in development of recombinant *C. glutamicum* strains for efficient lignocellulosic-biomass conversion to produce value-added chemicals and fuels.

**Electronic supplementary material:**

The online version of this article (doi:10.1186/s12934-016-0420-z) contains supplementary material, which is available to authorized users.

## Background

Advances in metabolic engineering and synthetic biology have opened-up opportunities for us to engineer microbial hosts to produce a range of industrially-relevant chemicals and fuels [1, 2]. In addition, oligonucleotide-mediated or CRISPR-CAS9-mediated genome editing technologies have been used to accelerated genomic evolution and enhance development of new strains [3, 4]. More efficient utilization of hexose and pentose derived sugars from lignocellulosic biomass (cellulose: ~48 %, xylan: ~22 %, lignin: ~25 %) [5] is advantageous to achieve economically-attractive bioprocesse for improving titers, productivities, and yields of value-added chemicals.

An industrial amino acid producer, *Corynebacterium glutamicum* [6] showed a broad range of sugar utilization such as hexose (i.g. glucose and gluconate) and disaccharide (i.g. maltose and sucrose) but some pentose (arabinose but xylose) [7]. Since there are great potentials of *C. glutamicum* as a microbial cell factory to produce other commercially relevant chemicals and fuels [8–10] from renewable lignocellulosic biomass, efficient utilization of cellulosic sugars is an inevitable goal. *C. glutamicum* has been successfully engineered for cell growth and production of biochemical using pentose sugars via either the heterologous xylose-isomerase pathway [11, 12] or Weimberg pathway [13]. For cellobiose utilization in *C. glutamicum*, recent genome sequencing of *C. glutamicum* R strain showed possible gene clusters that encode for functional EII permeases of PTS (BglF1 and BglF2), and for functional phospho-β-glucosidases (BglG1 and BglG2) [14]. Thus, an adaptive strain of *C. glutamicum* R strain grown in minimal medium with 0.2 % cellobiose and glucose has revealed a single-substitution mutation BglF(V217A or V317 M) for cellobiose utilization [14, 15]. The *C. glutamicum* R-CEL strain has been shown to utilize cellobiose, glucose and xylose simultaneously, but only possible under anaerobic conditions [16].

However, *C. glutamicum* ATCC 13032 is incapable of PTS-dependent utilization of methyl β-D-glucoside and cellobiose because it does not have any genes annotated to β-glucosidases (bG). In addition, no genes encoding for cellobiose-specific PTS permease were annotated [17]. To metabolize cellobiose, *C. glutamicum* ATCC 13032 must have an enzyme that cleaves the β-(1 → 4)-glycosidic linkage of cellobiose. Thus, bG-displaying or secreting *C. glutamicum* strains have been developed and exhibited the complete consumption of 20 g/L cellobiose in 4 days with l-lysine production [18]. The cellobiose utilization was quite slow, compared to the glucose consumption of current *C. glutamicum* strains. Thus, optimizing gene expression and maximizing the activity of bG was necessary for better production of l-lysine and other chemicals. Recently, *Saccharomyces cerevisiae* (a cellobiose- and xylose-negative yeast strain) was engineered for cellobiose utilization by expressing cellodextrin transporter (CDT-1) and intracellular bG along with a xylose-consuming pathway in order to resolve carbon catabolite repression by glucose in hydrolysates [19]. This engineering has enabled simultaneous utilization of cellobiose and xylose as a model hydrolysate, and increased the productivity of ethanol.

**Fig. 1:**
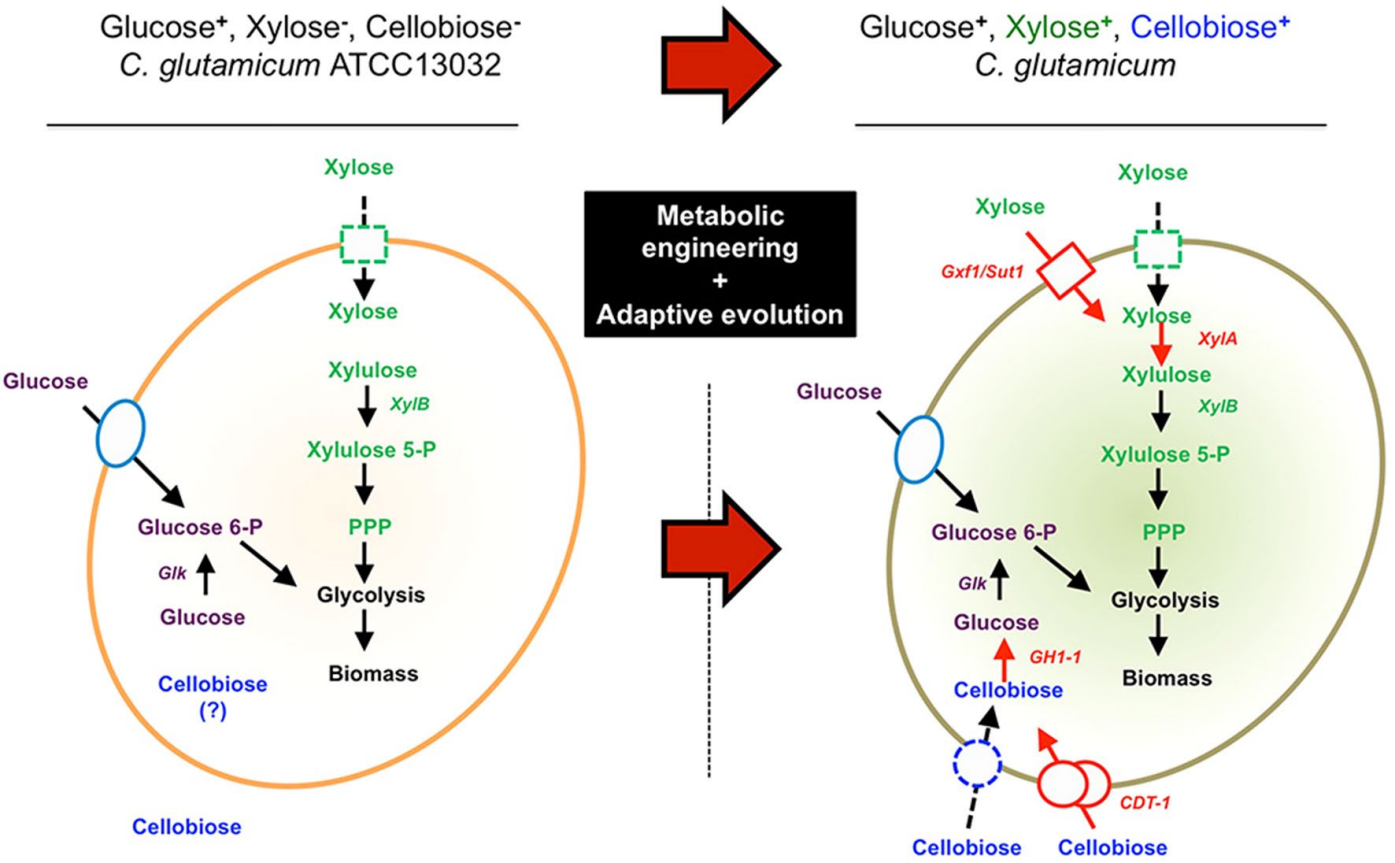
Scheme of reconstruction of cellobiose-utilizing and xylose-utilizing pathway in *C. glutamicum* ATCC 13032. *C. glutamicum* wild-type is not able to utilize cellobiose and xylose as sole carbon source (*left*). No genes for xylose isomerase, cellobiose transporters, and β-glucosidase are annotated (shown as *no arrow*). Through metabolic engineering and adaptive evolution of *C. glutamicum* strains (*right*), the cells were able to utilize cellobiose and xylose. Extracellular cellobiose was transported and intracellular β-glucosidase encoded by the *gh1*-*1* gene hydrolyzed intracellular cellobiose to glucose, which further was metabolized by glucokinase (Glk) into glycolysis. Xylose metabolic pathway consists of heterologous xylose isomerase (*xylA* from *E. coli*) and additional xylulose kinase (*xylB* from *E. coli*). Transporter of Gxf1 (*Candida intermedia*) and Sut1 (*Pichia stipitis*), respectively was introduced as a xylose transporter

Like *S. cerevisiae*, *C. glutamicum* ATCC 13032 is not able to utilize either cellobiose or xylose as sole carbon source. First, we performed metabolic engineering of *C. glutamicum* for cellobiose utilization by expressing a cellodextrin transporter and an intracellular bG (Fig. 1) and evolved the strains for the efficient cellobiose utilization. Subsequently, NGS-genomic and DNA microarray transcriptomic analysis were performed to characterize the evolved strains. Next, we have introduced the xylose-isomerase pathway [11, 12, 20] to the cellobiose-utilizing *C. glutamicum* strain for co-utilization of cellobiose and xylose. Our cellobiose-utilizing engineered strains were used to ferment the cellobiose and glucose derived from Canadian Ponderosa Pine in simultaneous saccharification and fermentation (SSF). Canadian Ponderosa Pine was used as a model lignocellulosic biomass.

## Results and discussion

### Utilization of cellobiose in *C. glutamicum* using metabolic engineering and adaptive evolution

**Fig. 2:**
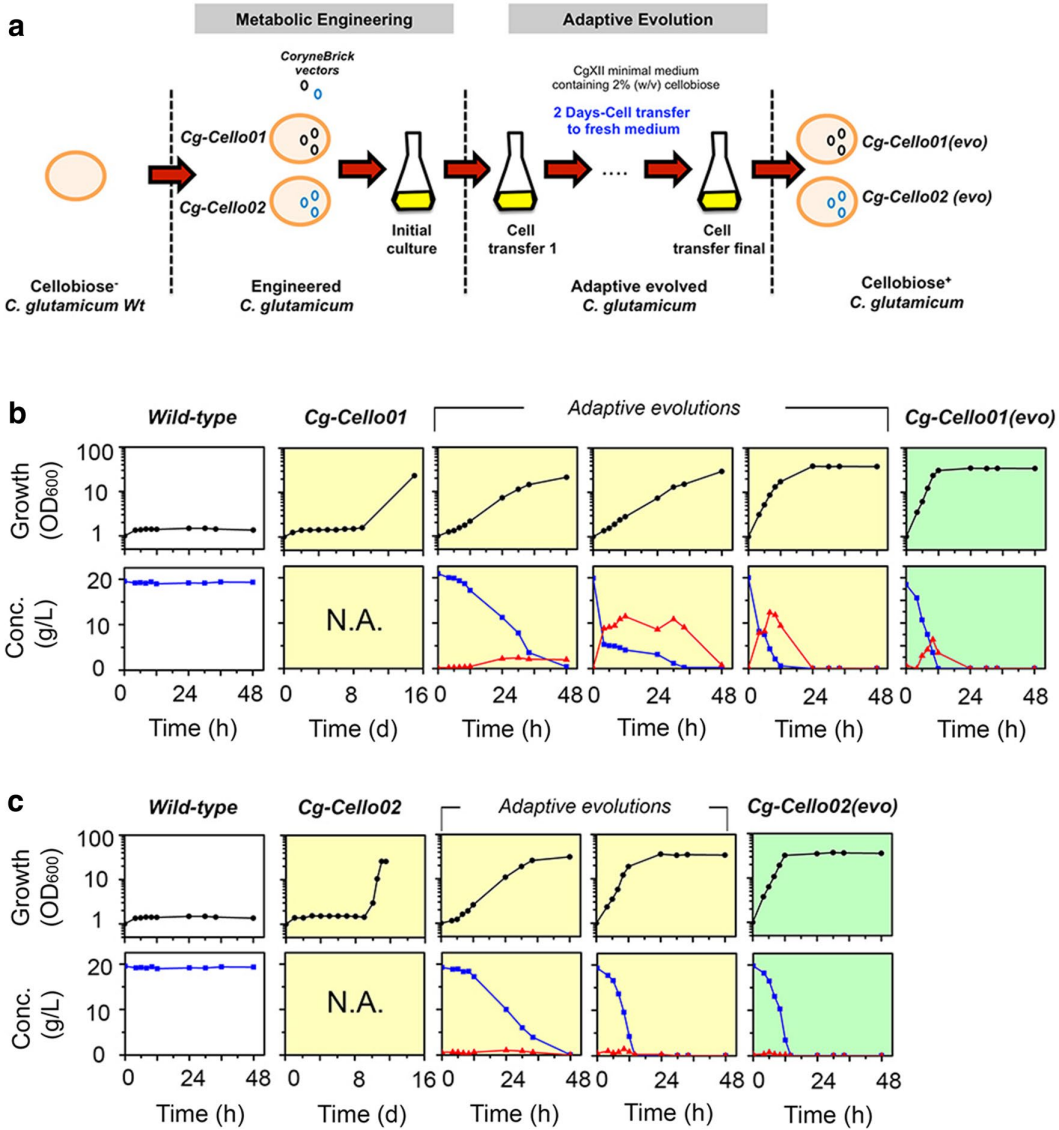
Adaptive evolution of engineered *C. glutamicum* strains. **a** Scheme of metabolic engineering and adaptive evolution of *Cg*-*Cello01* and *Cg*-*Cello02* strains were described. CoryneBrick vectors containing the *cdt*-*1* and/or *gh1*-*1* gene(s) were introduced into *C. glutamicum* wild-type, in which no growth and consumption of cellobiose were observed (**b** and **c**; the *first column*). Growth of *Cg*-*Cello01* and *Cg*-*Cello02* strain was not appeared initially. However, the maximal cell growths of *Cg*-*Cello01* and *Cg*-*Cello02* were observed after 16 d (**b**; the *second column*) and after 11 d (**c**; the *second column*), respectively. Subsequently, serial cell transfers were performed for adaptive evolutions of *Cg*-*Cello01* and *Cg*-*Cello02* in 48 h (**b** and **c**). Finally, *Cg*-*Cello01(evo)* and *Cg*-*Cello02(evo)* were obtained since growth and cellobiose consumption were unchanged. Growth at OD_600_, cellobiose (g/L) and glucose (g/L) were shown in a symbol of circle (*black*), square (*blue*) and triangle (*red*), respectively. *Data* represents mean values of at least three cultivations. (N.A.) not available

To test whether heterologous expressions of either CDT-1 transporter and bG or bG alone allow utilization of cellobiose in *C. glutamicum*, the *N. crassacdt*-*1* and *gh1*-*1* gene were codon-optimized and introduced into a CoryneBrick vector [11], pBbEB1c (Table 1). We attempted to grow *Cg*-*Cello01* strain containing both the *cdt*-*1* and *gh1*-*1* genes, and *Cg*-*Cello02* containing the *gh1*-*1* gene in CgXII minimal medium containing 2 % (w/v) cellobiose as sole carbon source. No growth of *Cg*-*Cello01* was observed for 4 d. Surprisingly, the cultures of *Cg*-*Cello01* strain exhibited the growth only after day 16 (Fig. 2). As soon as we observed the maximal cell growth of each strain (corresponding to the growth in presence of 2 % glucose), the adapted strain was transferred to fresh CgXII minimal medium containing 2 % (w/v) cellobiose for 48 h. After the first transfer, the growth and residual sugars in the culture medium were determined for each cell culture. However, no cell growth was observed for *Cg*-*pBbEB1c* as a control. During the adaptive evolution of *Cg*-*Cello01* strain by three serial transfers, cellobiose was gradually consumed during cell cultures (Fig. 2b). Interestingly, glucose in the medium was detected up to 12 g/L during the evolution and then glucose derived from cellobiose was consumed after cellobiose was completely depleted. In the last evolutionary round (third serial transfer), *Cg*-*Cello01(evo)* showed the complete cellobiose consumption in 12 h and glucose (5 g/L) was minimally secreted.

**Table 1:** Bacteria strains and plasmids used in this study

Strain or plasmid	Relevant characteristics	Source or reference
Strains		
* E. coli* HIT-DH5α	F^−^(80d *lac*Z M15) (*lac*ZYA-*arg*F) U169 *hsd*R17(r^−^ m^+^) *rec*A1 *end*A1 *rel*A1 *deo*R96	[38]
* C. glutamicum* ATCC 13032	*C. glutamicum* wild-type strain (ATCC 13032)	ATCC
* Cg*-*pBbEB1c*	*C. glutamicum* wild-type harboring pBbEB1c, Cm^r^ CoryneBrick empty vector	[39]
* Cg*-*EcXylAB*	*C. glutamicum* wild-type harboring pBbEB1c-*EcXylA*(cg.co) -*EcXylB*(cg.co), Cm^r^	[11]
* Cg*-*Cello01*	*C. glutamicum* wild-type harboring pBbEB1c-CT-bG, Cm^r^	This study
* Cg*-*Cello02*	*C. glutamicum* wild-type harboring pBbEB1c-bG, Cm^r^	This study
* Cg*-*Cello01(evo)*	Cellobiose-adapted *Cg*-*Cello01* strain, Cm^r^	This study
* Cg*-*Cello02(evo)*	Cellobiose-adapted *Cg*-*Cello02* strain, Cm^r^	This study
* Cg*-*Evo1*	Cellobiose-adapted *Cg*-*Cello01* strain after plasmid curing (plasmid-free), cellobiose^−^, Cm^s^	This study
* Cg*-*Evo2*	Cellobiose-adapted *Cg*-*Cello02* strain after plasmid curing (no plasmids), cellobiose^−^, Cm^s^	This study
* Cg*-*Cello03*	*Cg*-*Evo1* harboring pBbEB1c-bG, Cm^r^	This study
* Cg*-*Cello04*	*Cg*-*Evo2* harboring pBbEB1c-bG, Cm^r^	This study
* Cg*-*Cello03*-*Xyl01*	*Cg*-*Cello03* harboring pBbEB1c-*bG*-*XIK*, Cm^r^	This study
* Cg*-*Cello03*-*Xyl02*	*Cg*-*Cello03* harboring pBbEB1c-*bG*-*XIK*-*XTg,* Cm^r^	This study
* Cg*-*Cello03*-*Xyl03*	*Cg*-*Cello03* harboring pBbEB1c-*bG*-*XIK*-*XTs,* Cm^r^	This study
* Cg*-*Cello04*-*Xyl01*	*Cg*-*Cello04* harboring pBbEB1c-*bG*-*XIK*, Cm^r^	This study
* Cg*-*Cello04*-*Xyl02*	*Cg*-*Cello04* harboring pBbEB1c-*bG*-*XIK*-*XTg,* Cm^r^	This study
* Cg*-*Cello04*-*Xyl03*	*Cg*-*Cello04* harboring pBbEB1c-*bG*-*XIK*-*XTs,* Cm^r^	This study
Plasmids		
pBbEB1c	ColE1 (Ec), pBL1 (Cg), Cm^r^, P_*trc*_, BglBrick sites, CoryneBrick vector	[39]
pBbEB1c-CT-bG	pBbEB1c derivative containing each codon-optimized *N. crassa cdt1* and *gh1*-*1* genes	This study
pBbEB1c-bG	pBbEB1c derivative containing each codon-optimized *N. crassa gh1*-*1* gene	This study
pBbEB1c-bG-XIK	pBbEB1c derivative containing each codon-optimized *N. crassa gh1*-*1* gene and *E. coli xylA* and *xylB* genes	This study
pBbEB1c-bG-XIK-XTg	pBbEB1c derivative containing each codon-optimized *N. crassa gh1*-*1* gene and *E. coli xylA* and *xylB* genes and *C. intermedia gxf1* gene	This study
pBbEB1c-bG-XIK-XTs	pBbEB1c derivative containing each codon-optimized *N. crassa gh1*-*1* gene and *E. coli xylA* and *xylB* genes and *P. stipitis sut1* gene	This study

On the other hand, fewer adaptations of *Cg*-*Cello02* were performed to obtain *Cg*-*Cello02(evo)*. *Cg*-*Cello02* strains exhibited the growth after day 11. Only twice serial transfers were performed to obtain the desired phenotype, of which the strain completely consumed its cellobiose in 12 h. No glucose derived from cellobiose was secreted during the adaptive evolution rounds (Fig. 2c). Finally, we confirmed no phenotypic changes (cell growth and cellobiose consumption) shown over more than twenty’s serial transfers into fresh medium (not shown) after *Cg*-*Cello01(evo)* and *Cg*-*Cello02(evo)* were obtained. As a result, the patterns of cell growth and 2 % (w/v) sugar consumption of *Cg*-*Cello01(evo)* and *Cg*-*Cello01(evo)* were almost identical to those of the wild-type [11], regardless of cellobiose or glucose. Finally, we obtained *Cg*-*Cello01(evo)* and *Cg*-*Cello02(evo)* strains as cellobiose-utilizing *C. glutamicum* ATCC 13032 derivative strains. *Cg*-*Cello01(evo)* strain is the fastest cellobiose-utilizing strain known under aerobic conditions.

**Fig. 3:**
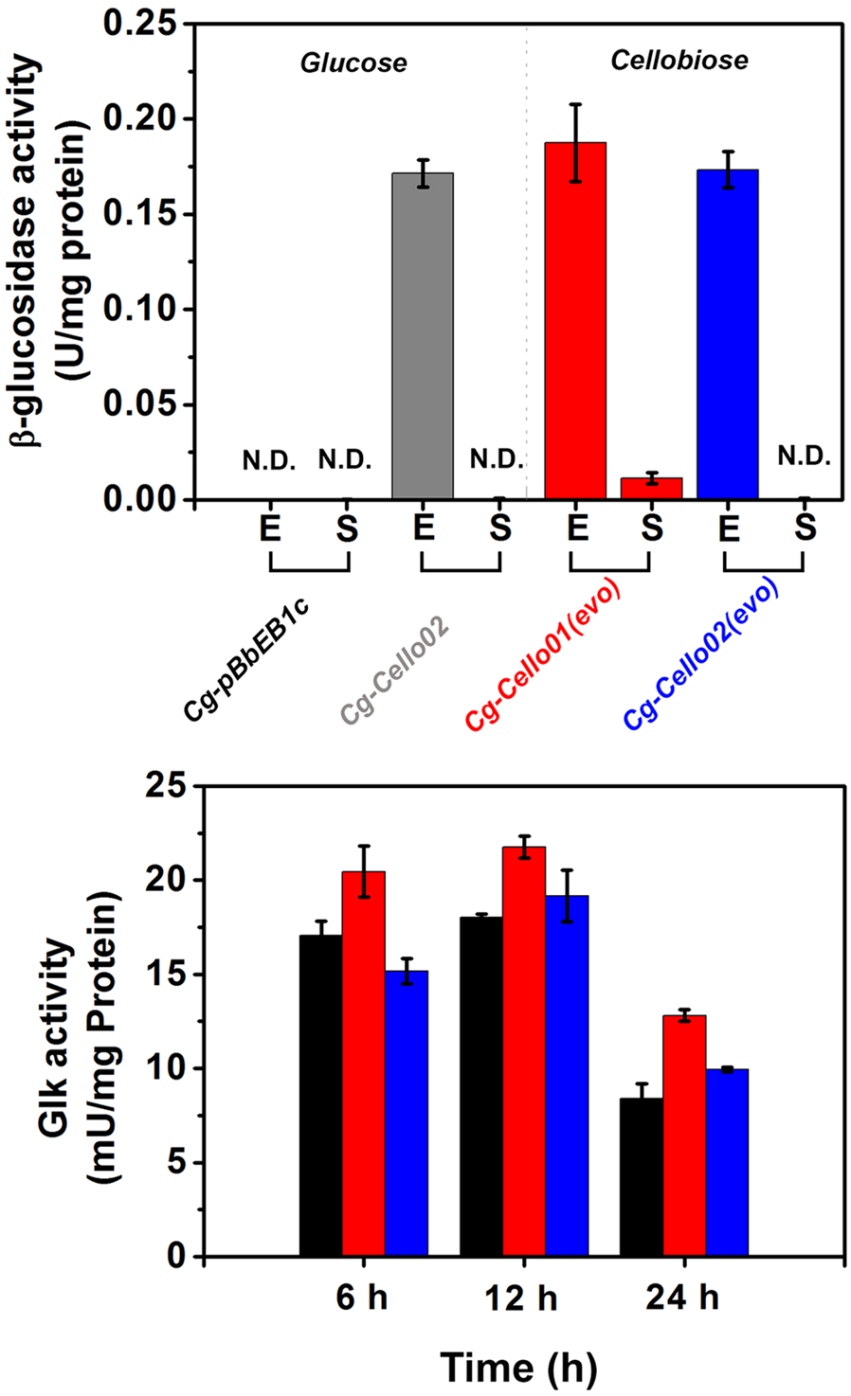
Measurement of β-glucosidase (bG) and glucokinase (Glk) activities in *C. glutamicum* wild-type or evolved strains. The *Cg*-*pBbEB1c* (*black*) and *Cg*-*Cello02* (*grey*) were cultivated in CgXII medium with 2 % (w/v) glucose. The evolved *Cg*-*Cello01(evo)* (*red*) and *Cg*-*Cello02(evo)* (*blue*) strains were cultivated in CgXII medium with 2 % (w/v) cellobiose. The cell extract (E) and cell-free supernatant (S) were used for the measurement of the bG activities (U/mg protein) when the strains were cultivated for 24 h (*upper panel*). The cell extracts from the strains grown for 6, 12, or 24 h were used for the measurement of the Glk activities (mU/mg protein) (*lower panel*). *Data* represents mean values of at least three cultivations and *error bars* represent standard deviations. (N.D.) not detected

Cellobiose-utilizing *C. glutamicum* requires activities of bG and glucokinase (Glk) for the catabolism of cellobiose in the cytosol. Thus, we checked whether the bG and Glk activities of the *Cg*-*Cello01(evo)* and *Cg*-*Cello02(evo)* strains were altered (Fig. 3). As a result, the control (*Cg*-*pBbEB1c*; the wild-type carrying the empty vector) showed no bG activity (both cell extract and supernatant). On the other hand, levels of the bG activities in the cell extracts of *Cg*-*Cello01(evo)* and *Cg*-*Cello02(evo)* grown on 2 % cellobiose were measured at 0.2 ± 0.01 and 0.17 ± 0.002 U/mg, respectively (Fig. 3). Significantly low or none of the bG activities were seen in the cell-free supernatants from *Cg*-*Cello01(evo)* or *Cg*-*Cello02(evo)* cultures. Also, the bG activity levels were quite similar to a bG activity from wild-type expressing GH-1-1 alone (initial *Cg*-*Cello02*) was grown on 2 % glucose, the bG activity (0.17 ± 0.01). Thus, these results indicated that the adaptive evolution did not alter the intracellular expression of heterologous bG. Also, no exogenous expression of bG was occurred due to possible genetic mutations. In addition, we measured Glk activity over the culture interval until the carbon sources were depleted. The Glk activity of *Cg*-*Cello01(evo)* and *Cg*-*Cello02(evo)* was not significantly different the Glk activity from *Cg*-*pBbEB1c*. Therefore, having bG activity in the cytosol of *C. glutamicum* is one of key steps to utilization of cellobiose, but increasing or high bG activity is not necessary for better cellobiose utilization.

### Characterization of the evolved cellobiose-positive *C. glutamicum* strains

Through metabolic engineering and adaptive evolution of *C. glutamicum*, we obtained the cellobiose-utilizing strains, *Cg*-*Cello01(evo)* and *Cg*-*Cello02(evo)*. Since *C. glutamicum* wild-type does not have any genes annotated cellobiose transporter, we investigated if the evolved cells have either functional CDT-1 transporters or altered transporters for the uptake of cellobiose.

First, plasmids used for metabolic engineering were isolated to characterize genetic mutations occurred during the adaptive evolution. When we compared the original sequence of plasmids, pBbEB1c-CT-bG and pBbEB1c-bG, in-frame deletion and a point mutation were found in the region of replication of origins (Additional file 1: Figure S1), but no mutations were found on the sequence of the *gh1*-*1* gene. Interestingly, the *cdt*-*1* gene that was present on the pBbEB1c-CT-bG in the *Cg*-*Cello01* strain was missing, which was confirmed by DNA sequencing. This could be due to intra-molecular recombination that occurs during the adaptive evolution at the identical and ribosomal binding synthetic DNA sequences of the *cdt*-*1* and *gh1*-*1* genes. Also, the gel images of the *cdt*-*1* gene by colony PCR were shown for the loss of *cdt*-*1* gene during the evolutionary process of the *Cg*-*Cello01* to *Cg*-*Cello01(evo)* strains (Additional file 1: Figure S2). This result indicated that heterologous expression of CDT-1 was not suitable for the cellobiose uptake in *C. glutamicum*. Also, we found that there were no mutations found in the sequence of the *gh1*-*1* gene encoding for bG on the plasmids although a substitution mutation of BglF (V217A or V317 M) was found on the adaptive *C. glutamicum* R strain [15]. Thus, the plasmid sequencing results confirmed that the intracellular expression of heterologous bG was sufficient to utilize cellobiose as sole carbon source in *Cg*-*Cello01(evo)* and *Cg*-*Cello02(evo)* strains (Fig. 3).

To characterize the genetic basis of cellobiose-utilizing *C. glutamicum*, next-generation sequencing (NGS) analysis was applied to fully-evolved *Cg*-*Cello01(evo)* and *Cg*-*Cello02(evo)* strains, compared with the reference genome sequence of *C. glutamicum* ATCC 13032 (Additional file 1: Table S1 and Table S2). As a result, in the genome sequence of *Cg*-*Cello01(evo)* strain thirty-six genes were mutated with thirty-one single-nucleotide variants including missense (15 variants) and silent mutations (five variants) in the coding regions, two multi-nucleotide variants, one insert and two deletions (Additional file 1: Table S1). *Cg*-*Cello02(evo)* strain exhibiting shorter adaption were shown for more mutations occurred. Three hundred single-nucleotide variants including missense (123 variants), nonsense (six variants), silent mutations (98 variants) in the coding regions were identified along with 41 insertions and 28 deletions in the nucleotide sequence. Yet, the reasons for the high number of mutations for the evolved strains were unclear since the complete genome sequences of cellobiose-utilizing *C. glutamicum* R-CEL and CEL2 strains were not available [14, 15]. Specifically, multiple genetic variants were found in genes encoding for putative proteins, phage integrase, ATPase component of ABC-type transport system (Cg2184), GTPase, tranposase (Cg2461), and intergentic regions. Thus, we searched the identical genetic variants in between *Cg*-*Cello01(evo)* and *Cg*-*Cello02(evo)* strains. Finally, 10 identical genetic variants were identified (Table 2). Three genes encoding for membrane proteins (ABC-type transporter, RibX, LysE-type transloactor) were shown for the missense mutations, which could be responsible membrane proteins for the cellobiose uptake. In addition, two genes (*wzz* and *fruR*) involved in sugar metabolism were mutated, changing its amino acid sequences (Glu363Asp and Gly75Val), respectively. MiaB (tRNA methylthiotransferase (MiaB), Maltose-bninding protein (AmyE), and Benoate 1,2-dioxgenase (BenA) were also confirmed to be mutated. Unlike evolved *C. glutamicum* R strains, *C. glutamicum* ATCC1304 required multiple mutations for the cellobiose uptake in a concert of altered membrane proteins, metabolic and regulatory proteins, translational processing, and auxiliary proteins. The mechanism of those muteins for cellobiose utilization remains unclear.

**Table 2:** List of common mutations of *C. glutamicum* *Cg*-*Cello01(evo)* and *Cg*-*Cello02(evo)* strains

Reference position	Gene name	Type	Reference nucleotide	Allele nucleotide	Coding region change	Amino acid change	Annotation
32227	*cg0045*	MISSENSE	A	T	551A > T	Asn184Ile	Probable ABC transport protein, membrane component
364912	*cg0414*	MISSENSE	A	C	1089A > C	Glu363Asp	Wzz, cell surface polysaccharide biosynthesis/chain length determinant protein
1689677	*cg1796*	MISSENSE	C	T	103G > A	Glu35Lys	RibX, putative membrane protein-*C. ammoniagenes* RibX homolog
2041951	*cg2118* ^a^	MISSENSE	G	T	224G > T	Gly75Val	FruR, transcriptional regulator of sugar metabolism, DeoR family
2058943	*cg2135*	MISSENSE	A	G	490T > C	Ser164Pro	MiaB, tRNA methylthiotransferase
2296630	*cg2380*	Deletion	C	–	270delG	Gly90 fs	Hypothetical protein Cg2380
2331324	*cg2412*	MISSENSE	C	T	154C > T	Pro52Ser	Hypothetical protein Cg2412
2545730	*cg2637* ^b^	MISSENSE	G	A	436G > A	Asp146Asn	BenA, benzoate 1,2-dioxygenase alpha subunit (aromatic ring hydroxylation dioxygenase A)
2607484	*cg2705*	MISSENSE	G	A	407C > T	Ala136Val	AmyE, maltose-binding protein precursor
2826260	*cg2941*	MISSENSE	T	C	577A > G	Ile193Val	LysE type translocator

Full list of all mutations of *C. glutamicum* *Cg*-*Cello01(evo)* and *Cg*-*Cello01(evo)* strains were described in the Additional file 1: Table S1 and S2

^a^The mRNA expression of the *cg2118* gene was highly up-regulated

^b^The mRNA expression of the *cg2637* gene was highly down-regulated. See the details in the text

**Fig. 4:**
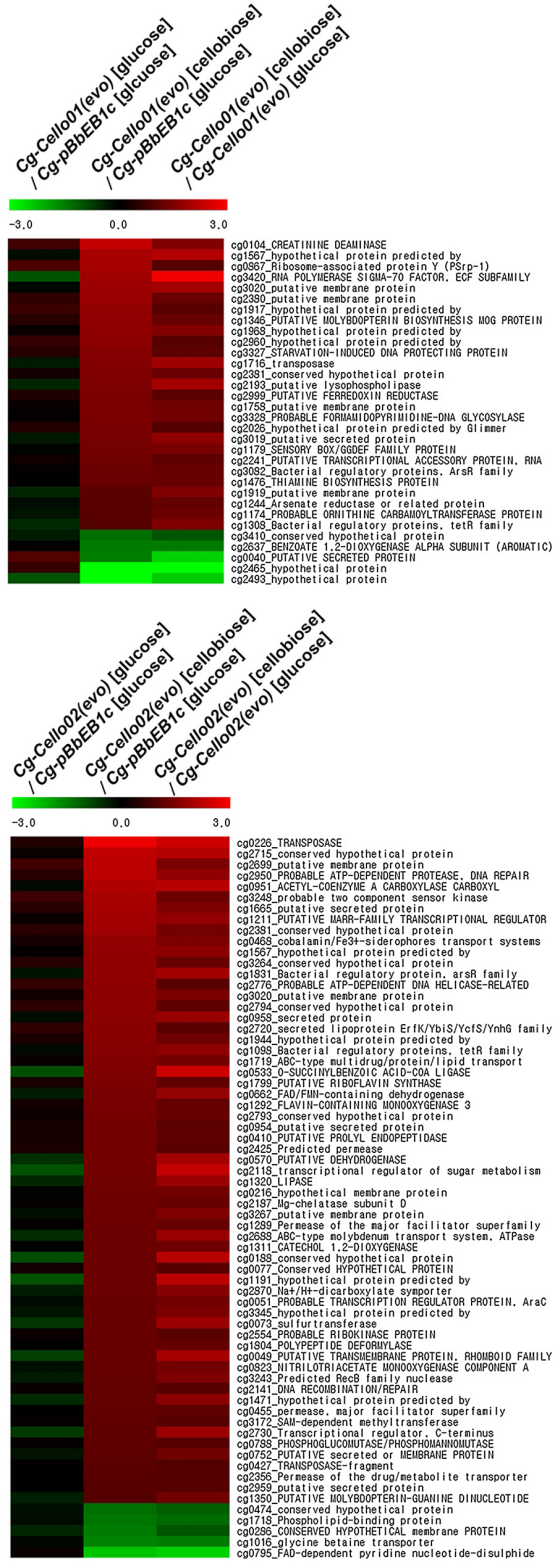
Heat map of altered gene expressions of *C. glutamicum* strains with cellobiose or glucose. Evolved *C. glutamicum* strains [*Cg*-*Cello01(evo)* and *Cg*-*Cello02(evo)*] grown on 2 % (w/v) cellobiose were tested with either a control (*Cg*-*pBbEB1c*) or *Cg*-*Cello01(evo)* and *Cg*-*Cello02(evo)* grown 2 % (w/v) glucose. The mRNA expression changed with 2-fold up- and 0.5-fold down-regulated were selected in the evolved strains with cellobiose over glucose (the *third columns*) as well as a control (the *second columns*). The mRNA ratios are averages from at least duplicated experiments. Heat maps generated by MeV (MutiExperiment Viewer ver. 4.8) showed differential gene expression of significantly changed genes. Up-regulated signals relative to the mean were colored in *red*. Down-regulated were colored in *green* (*scale bar*, log 2 of mRNA ratio). The criterion used for selection of RNA ratios was a signal-to-noise ratio of >3 for either Cy5 fluorescence. For the significantly changed genes, P < 0.05 as determined by a one-way ANOVA. The ID numbers of *C. glutamicum* were given at the *last column*, of which *data* were described in the Additional file 1: Table S1

Next, to characterize the genome-wide gene expression of the *Cg*-*Cello01(evo)* and *Cg*-*Cello02(evo)* strains utilizing cellobiose, we performed a DNA microarray-transcriptomic analysis to investigate whether the gene-expression levels of sugar transporters, or other membrane proteins, were differentially altered in the evolved strains during cellobiose consumption. We analyzed three groups: (1) gene expression of the evolved strains grown on glucose with a control (*Cg*-*pBbEB1c*) on glucose, (2) gene expression of the evolved strains grown on cellobiose with a control (*Cg*-*pBbEB1c*) on glucose, and (3) gene expression of the evolved strains grown on cellobiose with evolved strains on glucose. Those with gene expression that was 2-fold up- and 0.5-fold down-regulated in Group 2 and Group 3 were selected for further analysis (Fig. 4 and Additional file 1: Figure S3 and Table S3). Among 32 and 66 differentially altered genes for *Cg*-*Cello01(evo)* and *Cg*-*Cello02(evo)*, respectively, the gene expressions of four and fourteen membrane proteins (respectively) were changed significantly. Most of them are hypothetically annotated, according to the National Center for Biotechnology Information (NCBI) (accession no. NC003450).

In a comparison of transcriptomic analysis with next-generation sequencing-analysis, the mRNA expression of the regulon of FruR (the *ptsH*, *ptsF*, *pfkB1*, *ptsI* gene encoding for EII components of the PTS system) were not altered although the transcriptional FruR has been known to attenuate the induction of EII components of the PTS system in *C. glutamicum* R [21]. Thus, mutated FurR in the evolved strain may be not functional as a transcriptional regulator. However, the mRNA expression of a DeoR-type transcriptional regulator (FruR, Cg2118) was highly up-regulated (5-fold) in the *Cg*-*Cello02(evo)* strain in presence of cellobiose than glucose (Group 3). Also, the mRNA expression of benoate 1,2-dioxgenase (BenA) was only down-regulated (0.3-fold) in the *Cg*-*Cello02(evo)* strain in presence of cellobiose than glucose (Group 3), in which mRNA expression is repressed by global transcriptional regulator GlxR in sugar metabolism [22].

Since we observed the overexpressed hypothetical membrane proteins from the gene expression profiling of the cellobiose-utilizing strains (Fig. 4), we looked into the fatty acid profiles of cellular membranes of the wild-type strain, *Cg*-*Cello01(evo)*, and *Cg*-*Cello02(evo)*. Compared to the fatty acid profiles of the wild-type, lower palmitic acids (C_16:0_) and higher unsaturated steric acids (C_18:1*w9c*_) were measured in the lipids of the cellobiose-utilizing strains (*Cg*-*Cello01(evo)* and *Cg*-*Cello01(evo)*) (Table 3). This altered lipid profile of *C. glutamicum* was also shown for the wild-type growing on sodium oleate, and for mutants [23]. Moreover, cellobiose utilization influenced the fatty acid profiles of a recombinant *Rhodococcus opacus* PD630 expressing bG, which accumulated fatty acids from cellobiose [24]. Multiple mutations metabolic and regulatory proteins, translational processing, and auxiliary proteins, intergenic regions in evolved *C. glutamicum* could be responsible for altered lipid profiles.

**Table 3:** Fatty acid profiles of the cellobiose-utilizing *C. glutamicum* strains and the wild-type

Fatty acid composition (%)	*Cg*-*pBbEB1c*	*Cg*-*Cello01(evo)*	*Cg*-*Cello02(evo)*
C_10:0_	0.15	0.08	0.08
C_12:0_	0.25	–	–
C_12:0_ 3OH	–	0.06	0.05
C_14:0_	1.33	0.43	0.39
C_16:1_ w9c	0.84	0.51	0.46
C_16:0_	41.04	35.89	33.28
C_16:0_ 3OH	0.31	0.69	0.34
C_18:1_ w9c	54.24	59.97	63.4
C_18:0_	0.47	0.44	0.41
C_18:0_ 10-methyl	1.27	1.54	1.26
C_19:1_ iso I	0.09	0.33	0.33
C_19:1_ w6c/unknown fatty acids*	–	0.06	–

The *Cg*-*Cello01(evo) and Cg*-*Cello02(evo)* strains were cultivated with 2 % cellobiose as sole carbon source. The *Cg*-*pBbEB1c* stain was cultivated with 2 % glucose as sole carbon source. The fatty acid profiles of the *Cg*-*pBbEB1c* were almost identical to the profiles of the *C. glutamicum* wild-type [23]. Analysis of fatty acid and fatty acid methyl ester were followed by the standard protocol of Sherlock^®^ Microbial Identification System (MIS) of MIcrobial IDentification Inc. (MIDI)

Data represents mean values of duplicated cultivations

* Equivalent chain lengths (ECL) value = 18.846

Based on the NSG- and transcriptomic analysis, altered ABC-type transporters/hypothetical membrane proteins and sugar metabolism were responsible for efficient cellobiose utilization in *C. glutamicum*. However, it was difficult to pinpoint which single transporter is mainly designated for the cellobiose uptake. Rather, multiple gene mutations could be required for the efficient cellobiose uptake in *C. glutamicum*. Those mutated genes in common could be good targets for further engineering of *C. glutamicum* wild-type to explorer cellobiose uptake and corresponding sugar metabolism or protein structures. Thus, comprehensive next-generation sequencing-analysis could be required to analyze the evolving and evolved strains to investigate the most critical mutations for the cellobiose utilization.

### Reconstruction of cellobiose-positive chassis of the adaptive evolved *C. glutamicum* strains

Inverse engineering the *C. glutamicum* wild-type is necessary to construct rational microbial cells for cellobiose utilization. However, lack of multiple genome editing technology such as RNA-guided CRISPR-CAS9 [4] or MAGE [3] system of *C. glutamicum* has led to limitation of inverse engineering of *C. glutamicum* in this study. Thus, we decided to re-construct a cellobiose-positive chassis in which all multiple genetic changes were already reflected, for further engineering. We obtained plasmid-free strains by curing of plasmids in *Cg*-*Cello01(evo)* and *Cg*-*Cello02(evo)*, resulting in *Cg*-*Evo1* and *Cg*-*Evo2* (Table 1).

**Fig. 5:**
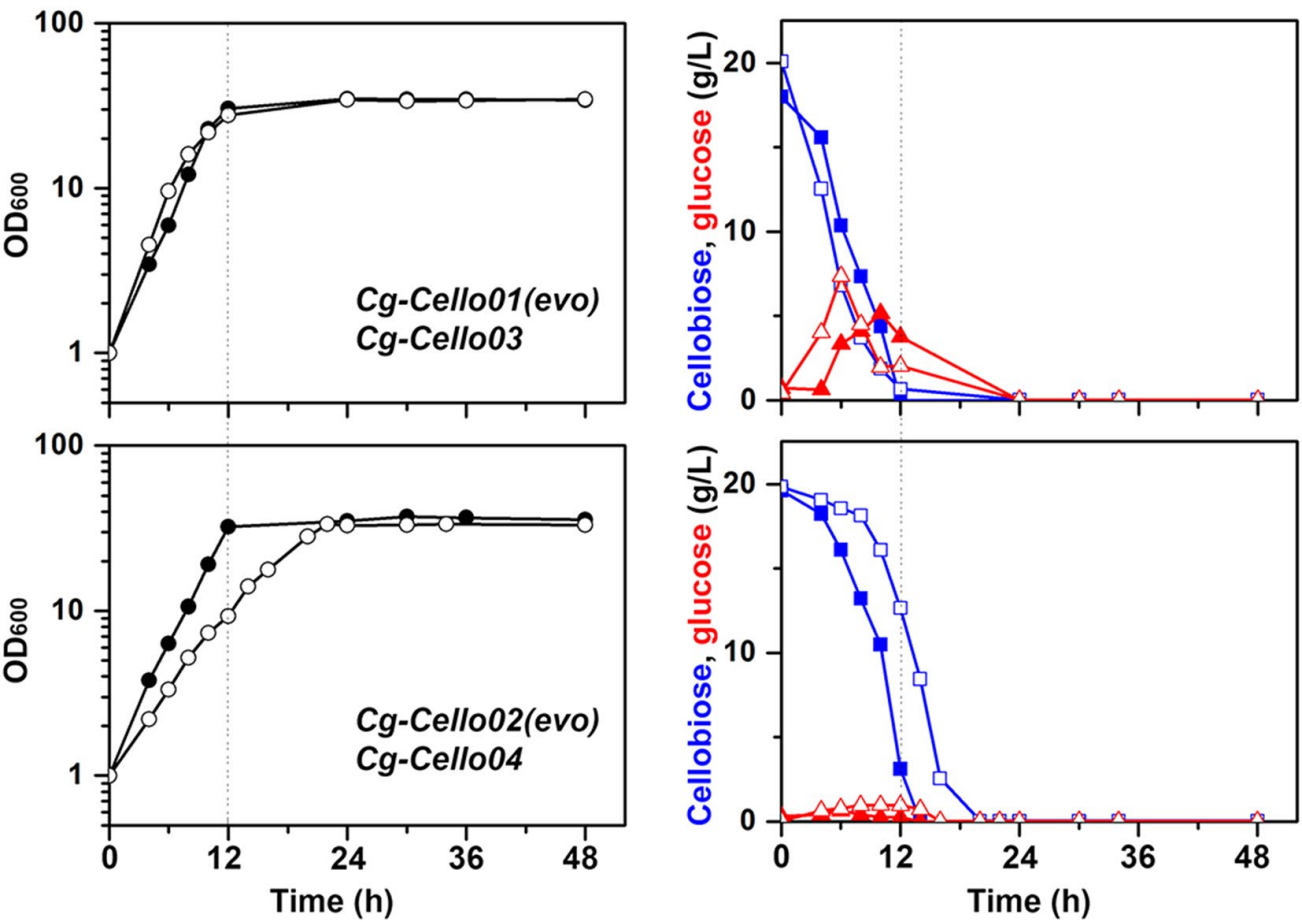
A comparison of cell growth and cellobiose consumption of evolved *C. glutamicum* strains. The evolved *C. glutamicum* strains was compared with reconstructed cellobiose-positive chassis of *C. glutamicum* strains. By plasmid-curing and re-transformation of pBbEB1c-bG plasmid, *Cg*-*Cello03* (*upper panels*; *open symbols*) and *Cg*-*Cello04* (*lower panels*; *open symbols*) strains as reconstructed cellobiose-positive chassis were obtained from *Cg*-*Cello01(evo)* (*upper panels*; *solid symbols*) and *Cg*-*Cello02(evo)* (*lower panels*; *solid symbols*) strains, respectively. The strains were cultivated in CgXII medium with 2 % (w/v) cellobiose as sole carbon source after the pre-cultivation in BHIS medium. Growth (*left panels*; *black circle*), cellobiose (*right panels*; *blue square*), glucose (*right panels*; *red triangle*) were shown. *Data* represents mean values of at least three cultivations

After the cultivation of *Cg*-*Evo1* and *Cg*-*Evo2* using 2 % cellobiose as sole carbon source, we confirmed that *Cg*-*Evo1* and *Cg*-*Evo2* did not grow at all. Thus, pBbEB1c-bG plasmid was introduced to *Cg*-*Evo1* and *Cg*-*Evo2*, yielding *Cg*-*Cello03* and *Cg*-*Cello04* strains (Fig. 5). *Cg*-*Cello03* and *Cg*-*Cello04* strains showed complete growth and consumption of cellobiose in the CgXII medium containing 2 % (w/v) cellobiose, without any adaptations or pre-culture with cellobiose (Fig. 5). This result supports that the *Cg*-*Evo1* and *Cg*-*Evo2* have already multiple genetic changes from its parental strain for the efficient cellobiose uptake.

When we compared the profiles of growth and cellobiose consumption, the *Cg*-*Cello03* strain showed almost identical patterns of growth and cellobiose consumption as its parental strain, *Cg*-*Cello01(evo)*. Glucose derived from cellobiose was secreted into the medium while both *Cg*-*Cello01(evo)* and *Cg*-*Cello03* strains consumed the cellobiose. In the case of *Cg*-*Cello04* strain, of which the parental strain is the *Cg*-*Cello02(evo)* strain, the rates of cell growth and cellobiose consumption were slightly retarded but no glucose was detected as for the parental strain. The reason for slower cellobiose consumption remains unclear.

Instead of inverse engineering, we successfully constructed cellobiose-positive *C. glutamicum* chassis strain that utilize cellobiose as sole carbon source under the conditions of aerobic culture by expressing intracellular bG alone. Moreover, the strains expressing intracellular bG exhibited better cellobiose utilization than any other strain either secreting bG into the medium, or displaying bG on the cell surface [18] in terms of the cellobiose consumption rate under aerobic conditions. Therefore, we obtained cellobiose-positive *C. glutamicum* chassis strains to perform metabolic engineering with cellobiose as sole carbon source.

### Co-utilization of cellobiose and xylose in *C. glutamicum* through metabolic engineering

**Fig. 6:**
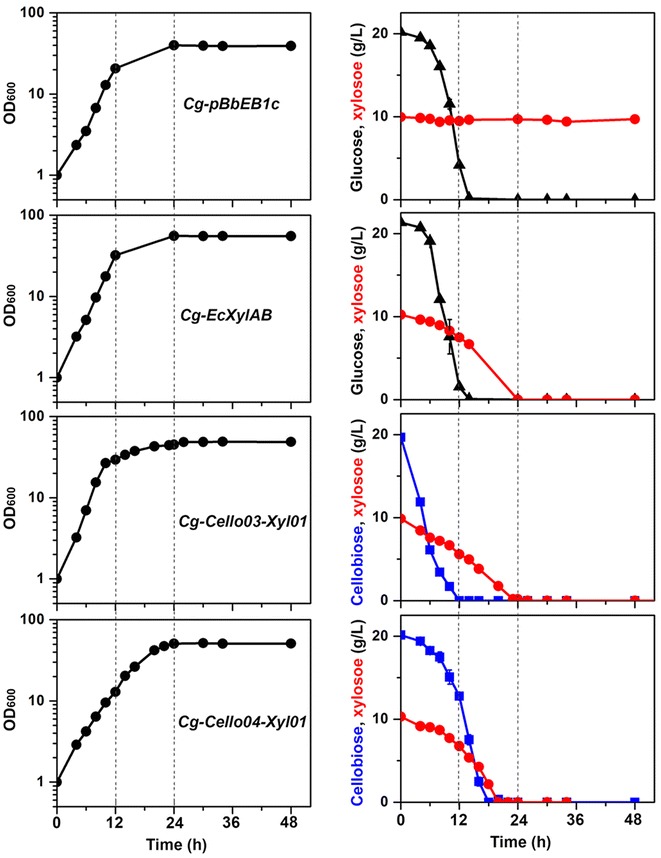
Co-consumption of cellobiose and xylose of engineered cellobiose-positive chassis of *C. glutamicum* strains. Two different cellobiose-negative strains (*Cg*-*pBbEB1c* and *Cg*-*EcXylAB*) and two different cellobiose-positive strains (*Cg*-*Cello03*-*Xyl01* and *Cg*-*Cello04*-*Xyl01*) co-expressing XylA and XylB were tested. The cellobiose-negative strains were cultivated in CgXII medium with a mixture of 2 % (w/v) glucose and 1 % (w/v) xylose. On the other hand, the cellobiose-positive strains were cultivated in CgXII medium with a mixture of 2 % (w/v) cellobiose and 1 % (w/v) xylose. Growth (*left panels*; *black circle*), cellobiose (*right panels*; *blue square*), glucose (*right panels*; *black triangle*), xylose (*right panels*; *red circle*) were shown. *Data* represents mean values of at least three cultivations and *error bars* represent standard deviations

Using the cellobiose-positive *C. glutamicum* chassis strains, we focused on co-utilization of xylose and cellobiose in *C. glutamicum* via direct cellobiose uptake and intracellular hydrolysis of cellobiose. As a result, *Cg*-*Cello03*-*Xyl01* and *Cg*-*Cello04*-*Xyl01* strains were able to co-utilize cellobiose and xylose under aerobic conditions (Fig. 6). Compared to the xylose consumption by *Cg*-*EcXylAB* in the presence of glucose, the xylose consumption rates of the engineered strains were improved in the presence of cellobiose, instead of glucose.

**Fig. 7:**
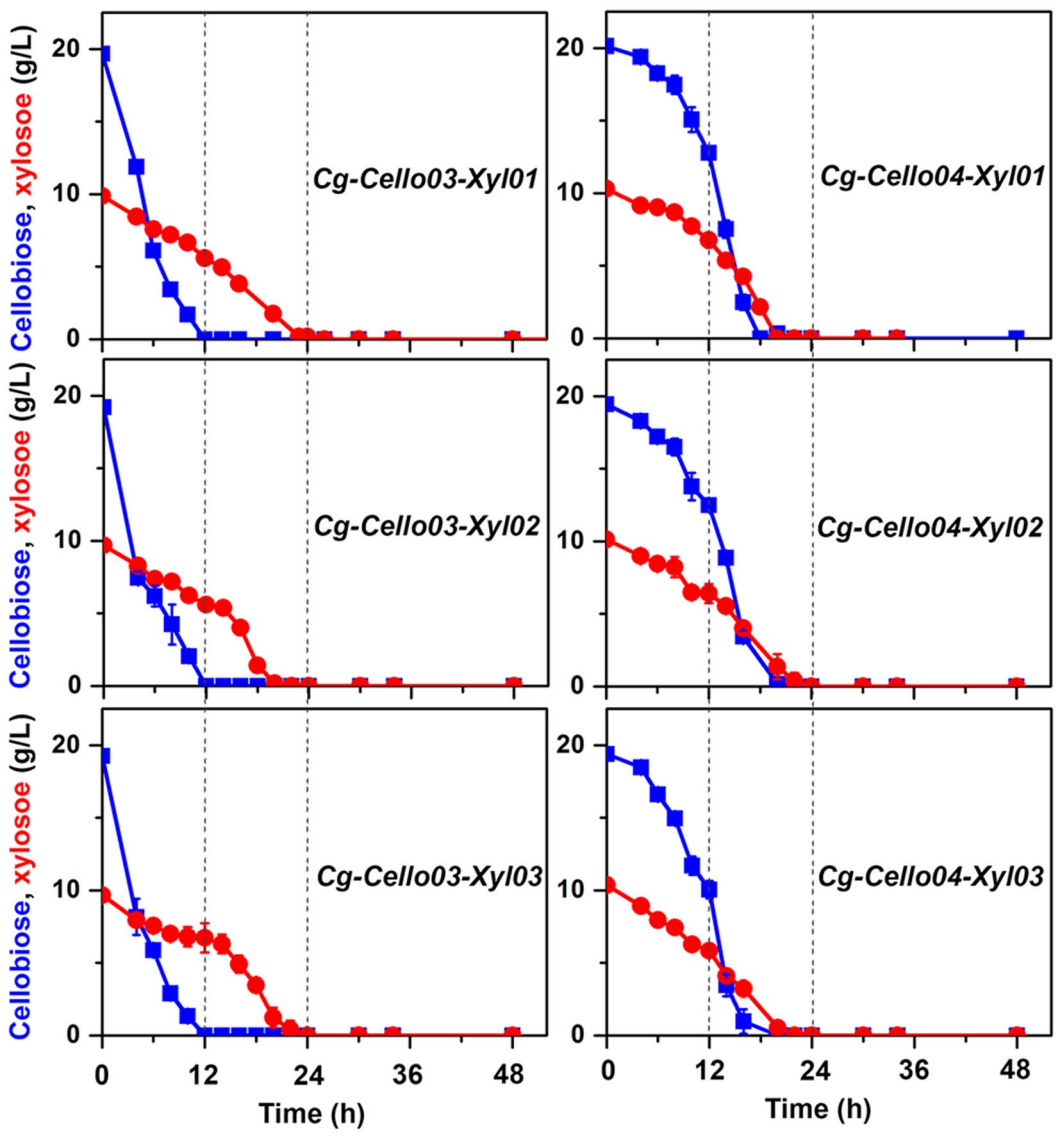
Additional sugar transporters for co-consumption of cellobiose and xylose. Two different cellobiose-positive strains [*Cg*-*Cello03*-*Xyl01* (*top left panel*) and *Cg*-*Cello04*-*Xyl01* (*top right panel*)] co-expressing XylA and XylB were further engineered with additional sugar transporters such as a Gxf1 (*Candia intermedia*) [29] and Sut1 (*Pichia stipites*) [30], yielding *Cg*-*Cello03*-*Xyl02* and *Cg*-*Cello03*-*Xyl03* (*left panels*) and *Cg*-*Cello04*-*Xyl02* and *Cg*-*Cello04*-*Xyl03* (*right panels*), respectively. The cellobiose-positive and xylose-positive strains were cultivated in CgXII medium with a mixture of 2 % (w/v) cellobiose and 1 % (w/v) xylose. Cellobiose (*blue square*), xylose (*red circle*) were shown. *Data* represents mean values of at least three cultivations and *error bars* represent standard deviations

As shown in the culture of *Cg*-*EcXylAB* strain with glucose and xylose, a biphasic growth of *Cg*-*Cello03*-*Xyl01* strain was observed after 12 h when cellobiose was completely consumed. However, a biphasic growth behavior was not shown by *Cg*-*Cello04*-*Xyl01* strain because the cellobiose was slowly consumed before 12 h, and cellobiose and xylose were almost simultaneously utilized and depleted in the culture between 12 and 24 h (Fig. 6). Moreover, *Cg*-*Cello04*-*Xyl01* strain reached higher optical density at first, compared to the *Cg*-*Cello03*-*Xyl01* strain. To further improve the engineered cellobiose- and xylose-utilizing strains, we introduced genes coding for sugar transporters (Gxf1 and Sut1) into *Cg*-*Cello03*-*Xyl01* and *Cg*-*Cello04*-*Xyl01* strains, respectively. But, the engineered strains with additional heterologous transporters did not show significant improvement of xylose utilization or co-utilization under the conditions of aerobic culture (Fig. 7).

Simultaneous consumption of cellobiose and xylose in engineered *S. cerevisiae* has resolved carbon catabolite repression and significantly increased ethanol productivity through co-fermentation [19]. Engineering of cellobiose-positive *C. glutamicum* also enabled the co-consumption of cellobiose and xylose (Figs. 6 and 7). However, compared to the cellobiose-utilizing *S. cerevisiae*, our cellobiose-positive *C. glutamicum* strains showed much faster cellobiose-consumption rates during aerobic culture. On the other hand, their xylose consumption rate was not much increased during co-fermentation. Expression of a pentose-specific transporter did not increase the rate, either. Therefore, we concluded that inefficient xylose utilization by *C. glutamicum* was another bottleneck for the co-fermentation of cellobiose and xylose although we did optimize the codon-usage of the corresponding *Escherichia coli* gene sequence [11]. Exploring an alternative xylose utilization pathway in *C. glutamicum* is necessary to provide faster xylose uptake [13, 20]. This strategy could then be further applied for co-fermentation of xylose and cellobiose.

### Hydrolysis of Canadian biomass and efficient SSF by engineered *C. glutamicum*

**Fig. 8:**
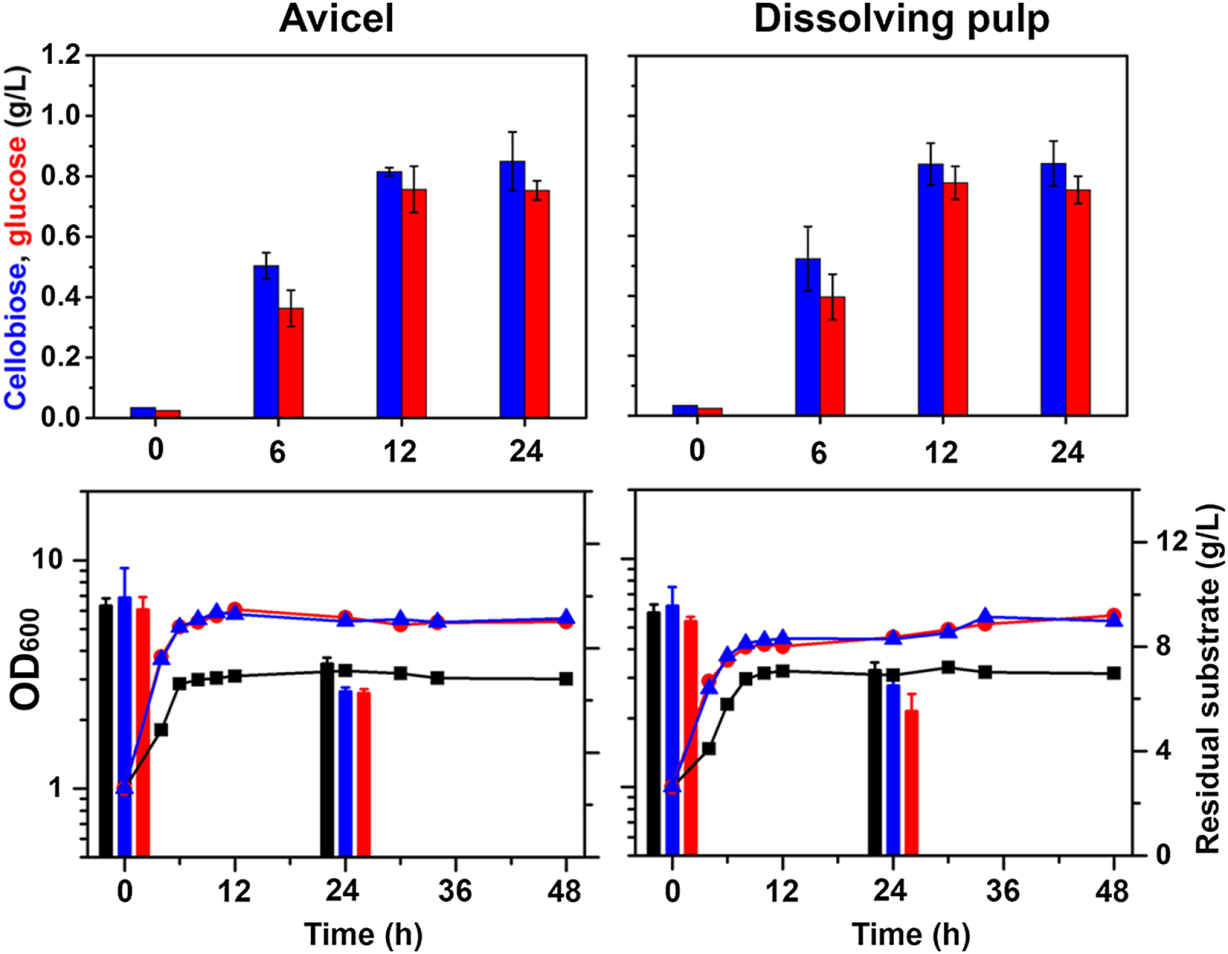
Profiles of conversion of Avicel^®^ PH-101 or DP by *C. glutamicum* strains. Celluclast 1.5 L (Sigma; Cat C2730) [75 filter paper unit (FPU)/g-glucan] was used as the cellulolytic enzymes for saccharification of Avicel^®^ PH-101 (*left panels*) or DP (*right panels*). For cellulolytic hydrolysis (*upper panels*), Avicel (1 % [w/v]) or DP (1 % [w/v]) were hydrolyzed at 30 °C and cellobiose (*blue bar*) and glucose (*red bar*) were measured. For SSF (*lower panels*), *Cg*-*pBbEB1c* (*black square*), *Cg*-*Cello03* (*blue triangle*) and *Cg*-*Cello04* (*red circle*) were cultivated with either Avicel (1 % [w/v]) or DP (1 % [w/v]) as sole carbon source in the presence of Celluclast 1.5 L and optical densities at 600 nm were measured after the sedimentation of the residual substrate (*lower panels*; *lines* and *symbols* with left Y-axis). For the measurement of the residual substrate (g/L), each residual substrate was measured at 0 and 24 h from the SSF cultures (*lower panels*; *bars* with right Y-axis). During SSF, no cellobiose and glucose were detected in the supernatant from the cultures. *Data* represents mean values of at least three cultivations and *error bars* represent standard deviations

Taking advantage of the capability for cellobiose utilization by two engineered *C. glutamicum* strains (*Cg*-*Cello03* and in *Cg*-*Cello04*), we applied the strains for efficient SSF of Canadian cellulosic hydrolysates. Before fermentation by *C. glutamicum*, we hydrolyzed either 1 % (w/v) Avicel^®^ PH-101, or 1 % (w/v) dissolving pulp (DP), with cellulase (Celluclast 1.5 L) under the same culture conditions (except for the cell type). As a result, equal amounts of cellobiose and glucose were detected for both cellulosic substrates (Fig. 8; upper panels), and similar conversion yields of total sugar (16.7 % and 16.15 %) were obtained for Avicel and DP, respectively. Also, enzymatic hydrolysis terminated after 12 h. There were not many differences between Avicel and DP as a cellulosic substrate for enzymatic hydrolysis with Celluclast 1.5 L.

Based on the enzymatic hydrolysis, we investigated whether the engineered strains (*Cg*-*Cello03* and in *Cg*-*Cello04*) were able to utilize cellulosic substrates during SSF. Thus, we cultivated 1 % (w/v) cellulosic substrate of either Avicel or DP as sole carbon source for the evolved *C. glutamicum* strains with cellulase. No lag phase was shown by either culture (Fig. 8; lower panels). Compared to a control (*Cg*-*pBbEB1c*; the wild-type with empty vector), the *Cello03* and *Cg*-*Cello04* strains showed faster growth, and reached nearly double growth at the end. When total amount of sugars was quantified independently, the total amount of sugars consumed in the *Cg*-*Cello03* and *Cg*-*Cello04* cultures were higher than that of a control. Moreover, we measured cellobiose and glucose in the supernatant, with the result that no cellobiose and glucose were detected during the culture of *Cello03* and *Cg*-*Cello04*. Therefore, the engineered *Cg*-*Cello03* and in *Cg*-*Cello04* strains were able to grow by simultaneously utilizing cellobiose and glucose from cellulosic hydrolysates although low conversion yields of cellulosic substrates limit further cell growth during SSF. Along with improvements of the enzymatic hydrolysis, the simultaneous saccharification and co-fermentation (SSCF) of pretreated plant biomass (hexose and pentose) could be accomplished using engineered *C. glutamicum* strains (*Cg*-*Cello03*-*Xyl01* and *Cg*-*Cello04*-*Xyl01*).

## Conclusions

Adaptive evolution of the microbial host to acquire desired environmental phenotypes was quite difficult unless growth-associated evolutions [25, 26]. In this study, integrated metabolic engineering and adaptive evolution allowed us to develop a cellobiose- and xylose-negative *C. glutamicum* strain that co-utilize cellobiose and xylose using. For further studies, we envision development of recombinant *C. glutamicum* strains based on the chassis strain, for efficient lignocellulosic-biomass conversion to create valuable products such as l-glutamate or l-lysine.

## Methods

### Bacterial strains, plasmids and culture conditions

All bacterial strains and plasmids used or constructed in this work are listed in Table 1. *E. coli* strains were grown in LB medium (containing per liter: 10 g tryptone, 5 g yeast extract, and 5 g NaCl) at 37 °C and 200 rpm. *C. glutamicum* ATCC 13032 and its derivatives were cultivated in BHIS medium (containing per liter: 37 g brain heart infusion, 91 g sorbitol) [27] at 30 °C and 200 rpm overnight and then incubated aerobically in CgXII medium (50 in 250 mL baffled Erlenmeyer flasks) [27] containing 2 % (w/v) cellobiose or a mixture of 2 % (w/v) cellobiose and 1 % (w/v) xylose supplemented with 25 μg/mL chloramphenicol at 30 °C on a rotary shaker at 200 rpm. All chemicals used in this study were purchased from Sigma-Aldrich (St. Louis, Mo). 0.5 mM isopropyl-β-D-thiogalactopyranoside (IPTG) was added for induction.

### Construction of plasmids and recombinant *C. glutamicum* strains

The *cdt*-*1* (NCU00801) and *gh1*-*1* (NCU00130) genes from *Neurospora crassa* [19] encoding for a cellodextrin transporter and a bG, respectively, were chosen for constructing a synthetic pathway in *C. glutamicum*. Each target gene was synthesized (Genscript, USA) with codon-optimization (Gene Designer 2.0 software [28]) for *C. glutamicum* and was assembled using a standard BglBrick cloning method, where the target gene is inserted at the *Eco*RI and *Xho*I sites of the CoryneBrick plasmid pBbEB1c [11]. Thus, the synthesized *gh1*-*1* gene was inserted, resulting pBbEB1c-bG. Subsequently, the synthesized *cdt*-*1* gene was placed in front of the *gh1*-*1* gene, resulting pBbEB1c-CT-bG. For utilization of xylose, the codon-optimized *xylA* and *xylB* genes from *E. coli* [11] were, subsequently, inserted to pBbEB1c-bG, resulting pBbEB1c-bG-XIK. In addition, the *gxf1* (*Candia intermedia*) [29] and *sut1* (*Pichia stipites*) [30] gene encoding for sugar-transporter were codon-optimized and inserted to pBbEB1c-bG-XIK, yielding pBbEB1c-bG-XIK-XTg and pBbEB1c-bG-XIK-XTs, respectively. Cloned DNA fragments were correctly verified by DNA sequencing.

The resulting plasmids were introduced into *C. glutamicum* by electroporation, and strain validation was performed by colony PCR [27]. The resulting strains are listed in Table 1.

### Adaptive Evolution of recombinant *C. glutamicum* strains

*Cg*-*Cello01* and *Cg*-*Cello02* strains were cultivated in CgXII minimal medium containing 2 % (w/v) cellobiose as sole carbon source. After the maximal cell growth of *Cg*-*Cello01* and *Cg*-*Cello02* were observed in 16 and 11 days, respectively, the cells were transferred to the fresh CgXII minimal medium containing 2 % (w/v) cellobiose, starting OD_600_ of 1 (Fig. 2a). Subsequently, the cells were transferred to the same fresh medium after every 48 h. Each culture of the cell was analyzed using HPLC to investigate the changes of the profiles of cellobiose utilization. The cell transfers were conducted until the rates of growth and cellobiose consumption were not changed, yielding *Cg*-*Cello01(evo)* and *Cg*-*Cello02(evo)* strains. *Cg*-*Cello01(evo)* and *Cg*-*Cello02(evo)* strains were further analyzed using DNA microarray. Plasmids from *Cg*-*Cello01(evo)* and *Cg*-*Cello02(evo)* strains were isolated and their DNA sequences were identified using partial overlapping primer-walking. In addition, plasmid-free strains were obtained by curing of plasmids in *C. glutamicum* as follows: after the electroporation of *Cg*-*Cello01(evo)* and *Cg*-*Cello02(evo)* strains, nonselective BHIS medium was inoculated at 30 °C. Each single colony was streaked onto BHIS agar plates either with or without chloramphenicol (25 μg/mL), yielding plasmid-free (Cm^s^) *Cg*-*Evo1* and *Cg*-*Evo2* strains, respectively. For co-utilization of cellobiose and xylose, the *xylA* (encoding for xylose isomerase) and *xylB* (encoding for xylulose kinase) genes was introduced into cellobiose-utilizing *Cg*-*Cello03* and *Cg*-*Cello04* strains, yielding *Cg*-*Cello03*-*Xyl01* and *Cg*-*Cello04*-*Xyl01* strains.

### HPLC analysis for glucose, xylose, and cellobiose quantification

For the measurement of the concentrations of glucose, xylose and cellobiose, culture supernatant was passed through a syringe filter (pore size of 0.2 μm) after centrifugation at 10,000*g* for 10 min. The concentrations of glucose and xylose were determined by high-performance liquid chromatography (HPLC system Agilent 1260, Waldbronn, Germany) equipped with a refractive index detector (RID) and an Aminex HPX-87 H Ion Exclusion Column (300 mm by 7.8 mm, Bio-Rad, Hercules, CA, USA) under the following conditions: sample volume of 20 μL, mobile phase of 5 mM H_2_SO_4_, flow rate of 0.6 mL/min, and column temperature of 65 °C.

### Enzymatic measurement of β-glucosidase and glucokinase activity

Recombinant strains were cultivated in CgXII medium containing 2 % (w/v) cellobiose but 2 % (w/v) glucose was used for the control (*Cg*-*pBbEB1c*). After incubation at 30 °C for 24 h, bG activities in the cell-free extracts or in the culture supernatants, respectively, were quantitatively measured in a 1 mL mixture containing 590 μL 500 mM potassium phosphate buffer (pH 7.0), 10 μL 500 mM MgCl_2_, 200 μL sample, 200 μL *p*-nitrophenyl-β-d-glucopyranoside (*p*NPG) as a substrate at 410 nm [16] (U; μmol of *p*NPG reduced min^−1^). For the determination of Glk activity (U/L) [31], the Glk activity in cell-free extracts was determined at 25 °C by measuring of the formation of NADPH at 340 nm in a coupled reaction containing 100 mM potassium phosphate buffer (pH 7.0), 20 mM glucose, 2 mM ATP, 25 mM MgCl_2_, 2 mM NADP and 2 U glucose-6-phosphate dehydrogenase (U; μmol of NADP reduced min^−1^).

### NGS-based genomic DNA sequencing analysis

Genomic DNAs of *Cg*-*Cello01(evo)* and *Cg*-*Cello02(evo*) were isolated from a single colony’s culture and purified using Wizard Genomic DNA purification kit (Promega, Cat.No. A1125). The genomes of *Cg*-*Cello01(evo)* and *Cg*-*Cello02(evo)* strains were sequenced using the Illumina Miseq 300 bp paired-end system (Illumina, Inc, San Diego, CA, USA) at ChunLab, Inc. (Seoul, South Korea). We obtained 5,753,368 reads of the genome to reach a 428.63-fold depth of coverage on *Cg*-*Cello01(evo)* and *Cg*-*Cello02(evo)*. The re-sequencing data were annotated by RNAmmer 1.2, tRNA scan-SE 1.21, Rapid Annotation using Subsystem Technology (RAST), Pipeline, and CLgenomics program (ChunLab, Inc). The detail procedures were described in the previous study [32].

### Transcriptomic analysis

Total RNA from *Cg*-*Cello01(evo)* and *Cg*-*Cello02(evo)* were sampled in the exponential phase. For Transcriptome analysis, extraction of total RNA and preparation of cDNA was followed by previous methods [33]. The cDNA probes were cleaned up using Microcon YM-30 column (Millipore, Bedford, MA) and then followed by Cy5-dye (Amersham Pharmacia, Uppsala, Sweden). The Cy5-labelled cDNA probes were cleaned up using the QIAquick PCR Purification Kit (Qiagen). Dried Cy5-labelled cDNA probes were re-suspended in hybridization buffer. Hybridization to a microarray slide (Corynebacterium_glutamicum 3 × 20 K) (Mycroarray.com, Ann Arbor, MI), staining, and scanning of the probe array were performed according to the manufacturer’s instructions. Hybridization image on the slide was scanned by Axon 4000B (Axon Instrument, Union City, CA). The analysis of the microarray data was performed using GenePix Pro 6.0 (Axon Instruments). The averages of the normalized ratios were calculated by dividing the average normalized signal channel intensity by the average normalized control channel intensity. All measurements were performed on duplicated technical replicates. Two independent samples were analyzed; their measurements were averaged for further analysis. The microarray data were deposited at the NCBI Gene Expression Omnibus, GEO under accession no. GSE65076 and at http://www.ncbi.nlm.nih.gov/geo/query/acc.cgi?acc=GSE65076.

### Fatty acids and lipid analysis

Fatty acid methyl esters were prepared as described previously [23, 34], and identified by gas chromatography with model 5898A microbial identification system (Microbial ID). Trimethylsilylated derivatives of fatty acids and methyl esters were analyzed by high-temperature gas chromatography on an HP 5790A gas chromatograph (Hewlett Packard), equipped with a flame-ionization detector on a 12 m high throughput screening (HTS) column with hydrogen gas as the carrier. Derivatives were identified by comparing their retention times to those of standards and by gas chromatography mass spectrometry analysis on a KRATOS MS50 spectrometer (ion source temperature set to 200 °C and ionization energy to 70 eV), respectively. For the analysis, colonies of *Cg*-*Cello01(evo) and Cg*-*Cello02(evo)* strains were obtained on CgXII agar plate containing 2 % cellobiose and colonies of *Cg*-*pBbEB1c* stain was obtained on CgXII agar plate containing 2 % glucose as.

### Cellulolytic hydrolysis of Avicel^®^ PH-101 and Canadian biomass and SSF by *C. glutamicum*

Avicel^®^ PH-101 (Sigma), microcrystalline cellulose and dissolving pulp (DP, pure cellulosic substrate less than 0.5 % lignin, less than 3 % xylan) [35] from Canadian Ponderosa Pine were used as substrate for cellulolytic hydrolysis and SSF by the cellobiose-utilizing *C. glutamicum* strains. Each cellulolytic hydrolysis and SSF was carried out in CgXII medium (pH 7.0) with 1 % (w/v) Avicel^®^ PH-101 or 1 % (w/v) dissolvping pulp at 30 °C and 200 rpm. Celluclast 1.5 L (Sigma; Cat C2730) [75 filter paper unit (FPU)/g-glucan] was used as the cellulolytic enzymes for saccharification of Avicel^®^ PH-101 or DP. Cellulase actitivity of the Celluclast 1.5 L was determined by the standard filter paper assay with the 3,5-dinitrosalicylic acid (DNS) method [36]. One unit of cellulose activity is defined as the amount of enzyme required to release 1 μmol of reducing sugar per mint at pH 4.8 and 50 °C. The enzyme activity of Celluclast 1.5 L was measured to be 28 FPU/mL. A colorimetric method based on the phenol–sulfuric acid reaction was used to determine the amount of residual substrate during SSF by quantifying total sugars [37].

### Availability of supporting data

The data set supporting the results of this article is available at NCBI GEO repository, [GSE65076 and http://www.ncbi.nlm.nih.gov/geo/query/acc.cgi?acc=GSE65076].

## Additional file

10.1186/s12934-016-0420-z Additional data and analysis for the evolved *C. glutamicum* strains.

## Abbreviations

bG: β-glucosidase
SSF: simultaneous saccharification and fermentation
HPLC: high-performance liquid chromatography
RID: refractive index detector
NGS: next-generation sequencing
GEO: gene expression omnibus
Glk: glucokinase
DP: dissolving pulp
SSCF: simultaneous saccharification and co-fermentation
