# Supplementary material for: Adaptive evolution and metabolic engineering of a cellobiose- and xylose- negative Corynebacterium glutamicum that co-utilizes cellobiose and xylose
Source: Microb Cell Fact. 2016 Jan 22;15:20. doi: 10.1186/s12934-016-0420-z (PMC4722713; doi:10.1186/s12934-016-0420-z)

Adaptive evolution and metabolic engineering of a cellobiose- and xylose-negative *Corynebacterium glutamicum* that co-utilizes cellobiose and xylose

Jungseok Lee[a], Jack N. Saddler[b], Youngsoon Um[a,c], Han Min Woo[a,c,d,*]

[a]Clean Energy Research Center, Korea Institute of Science and Technology, Hwarangro 14-gil 5, Seongbuk-gu, Seoul, Republic of Korea

[b]Department of Wood Science, University of British Columbia, Vancouver, BC V6T 1Z4, Canada.

[c]Department of Clean Energy and Chemical Engineering, Korea University of Science and Technology (UST), 217 Gajeong-ro, Yuseong-gu, Daejeon, Republic of Korea

[d]Green School (Graduate School of Energy and Environment), Korea University, 145 Anam-ro, Seongbuk-gu, Seoul, Republic of Korea

*Corresponding author at: Clean Energy Research Center, Korea Institute of Science and Technology (KIST), Seoul 136-791, Republic of Korea. Tel.: +82 2 958 5249; Fax: +82 2 958 5209. E-mail address: hmwoo@kist.re.kr (H.M. Woo).

Table S1. List of all mutations of C. glutamicum Cg-Cello01(evo)

Reference Position	gene name	Type	Reference	Allele	Count	Coding region change	Amino acid change	Annotation
**32227**	**cg0045**	**MISSENSE**	**A**	**T**	**213**	**n.551A>T**	**p.Asn184Ile**	**probable ABC transport protein, membrane component**
274977	cg0310	SILENT	T	C	233	n.654T>C		katA, catalase
**364912**	**cg0414**	**MISSENSE**	**A**	**C**	**233**	**n.1089A>C**	**p.Glu363Asp**	**wzz, cell surface polysaccharide biosynthesis / chain length determinant protein**
387460		SNV	T	C	183			
667963		SNV	C	T	245			
671706		Deletion	A	-	222			
736480	cg0822	MISSENSE	T	C	171	n.68T>C	p.Val23Ala	hypothetical protein cg0822
770809	cg0844	MISSENSE	G	A	190	n.3334C>T	p.Leu1112Phe	type II restriction enzyme, methylase subunit
1115810		MNV	CG	GT	58			
1279070	cg1368	MISSENSE	T	G	167	n.1383T>G	p.Asp461Glu	atpD, ATP synthase subunit B
**1689677**	**cg1796**	**MISSENSE**	**C**	**T**	**210**	**n.103G>A**	**p.Glu35Lys**	**ribX, putative membrane protein-C. ammoniagenes RibX homolog**
1751737	cg1859	MISSENSE	G	A	158	n.241G>A	p.Val81Ile	putative secreted protein
1757538		SNV	T	C	169			
2010622	cg2091	SNV	C	T	151			ppgK, polyphosphate glucokinase
2036724		SNV	A	C	171			
**2041951**	**cg2118**	**MISSENSE**	**G**	**T**	**165**	**n.224G>T**	**p.Gly75Val**	**transcriptional regulator of sugar metabolism, DeoR family**
**2058943**	**cg2135**	**MISSENSE**	**A**	**G**	**181**	**n.490T>C**	**p.Ser164Pro**	**miaB, trna methylthiotransferase**
2060507		SNV	C	T	181			
2083859	cg2166	SILENT	T	A	171	n.1332A>T		gpsI, putative polyribonucleotide phosphorylase / guanosine pentaphosphatesynthetase
2160737		SNV	C	T	142			
**2296630**	**cg2380**	**Deletion**	**C**	**-**	**150**	**n.270delG**	**p.Gly90fs**	**hypothetical protein cg2380**
**2331324**	**cg2412**	**MISSENSE**	**C**	**T**	**167**	**n.154C>T**	**p.Pro52Ser**	**hypothetical protein cg2412**
2425773	cg2517	SILENT	G	A	190	n.333C>T		hemN, coproporphyrinogen III oxidase
2509507	cg2599	SILENT	T	C	107	n.585T>C		pirin-related protein-fragment
**2545730**	**cg2637**	**MISSENSE**	**G**	**A**	**496**	**n.436G>A**	**p.Asp146Asn**	**benA, benzoate 1,2-dioxygenase alpha subunit (aromatic ring hydroxylation dioxygenase A)**
2556658		SNV	C	G	300			
**2607484**	**cg2705**	**MISSENSE**	**G**	**A**	**511**	**n.407C>T**	**p.Ala136Val**	**amyE, maltose-binding protein precursor**
2625489		MNV	TC	AG	58			
2703943		SNV	G	A	20			
2703945		Insertion	-	A	21			
2765908	cg2877	MISSENSE	G	A	204	n.229C>T	p.Arg77Cys	avtA, aminotransferase, uses alanine, keto-isovalerate and ketobutyrate
**2826260**	**cg2941**	**MISSENSE**	**T**	**C**	**197**	**n.577A>G**	**p.Ile193Val**	**lyse type translocator**
3043316	cg3161	MISSENSE	G	A	201	n.560G>A	p.Arg187Lys	hypothetical protein cg3161
3155328		SNV	T	C	194			
3156189		SNV	T	G	222			
3286754	cg3407	SILENT	G	A	157	n.132G>A		hypothetical protein cg3407

Note: Reference Position: Variant position on reference genome; Type: Variant type. SNV (single-nucleotide variant), MNV (multi-nucleotide variant), InDel(insertion or deletion); Reference: Reference nucleotide (ATGC) sequence at the position of the variant; Allele: The alleles found by the variant caller in the sequencing data; Counts: The number of reads supporting the alleles; For variants that fall within a coding region of a gene, the change is reported.;The report of synonymous or non-synonymous changes

Highlights: Common mutations of Cg-Cello1(evo) and CgCello2(evo)

Table S2. List of all mutations of C. glutamicum Cg-Cello02(evo)

Reference Position	gene name	Type	Reference	Allele	Count	Coding region change	Amino acid change	Annotation
1	cg0001	MISSENSE	G	C	29	n.1G>C	p.Val1Leu	dnaA, chromosomal replication initiation protein
2	cg0001	MISSENSE	T	A	21	n.2T>A	p.Val1Glu	dnaA, chromosomal replication initiation protein
3	cg0001	SILENT	G	T	29	n.3G>T	p.Val1Val	dnaA, chromosomal replication initiation protein
5	cg0001	MISSENSE	G	C	29	n.5G>C	p.Ser2Thr	dnaA, chromosomal replication initiation protein
7	cg0001	NONSENSE	C	T	27	n.7C>T	p.Gln3*	dnaA, chromosomal replication initiation protein
**32227**	**cg0045**	**MISSENSE**	**A**	**T**	**287**	**n.551A>T**	**p.Asn184Ile**	**probable ABC transport protein, membrane component**
48014	cg0062	MISSENSE	G	T	286	n.8C>A	p.Thr3Lys	ppp, protein phosphatase
66716	cg0085	Deletion	GT	G	404	n.19delT	p.Leu7fs	phoH1, ATPase related to phosphate starvation-inducible protein
66718	cg0085	MISSENSE	T	G	44	n.20T>G	p.Leu7Trp	phoH1, ATPase related to phosphate starvation-inducible protein
66719	cg0085	MISSENSE	G	T	35	n.21G>T	p.Leu7Phe	phoH1, ATPase related to phosphate starvation-inducible protein
268951		Insertion	C	CG	297			
273896	cg0309	SILENT	A	G	282	n.225T>C	p.Arg75Arg	sigC, RNA polymerase sigma factor
278749		SNV	A	T	271			
283203	cg0325	SILENT	A	G	273	n.115T>C	p.Leu39Leu	hypothetical protein cg0325
322773		SNV	TC	T	303			
**364913**	**cg0414**	**MISSENSE**	**A**	**C**	**365**	**n.1089A>C**	**p.Glu363Asp**	**wzz, cell surface polysaccharide biosynthesis / chain length determinant protein**
390755		Insertion	A	AG	313			
390759		SNV	T	G	30			
400382		Insertion	A	AG	318			
409175	cg0464	MISSENSE	G	T	325	n.800C>A	p.Ala267Asp	ctpA, copper-transporting ATPase
414930	cg0469	MISSENSE	C	A	41	n.218C>A	p.Ser73Tyr	cobalamin/Fe3+-siderophores transport system, ATPase component
461790	cg0517	MISSENSE	A	G	341	n.680A>G	p.Tyr227Cys	hemY, protoporphyrinogen oxidase
554147		Deletion	TG	T	357			
554256		Deletion	AG	A	345			
554304		Deletion	CG	C	333			
578119		Insertion	T	TG	352			
610990	cg0691	Insertion	A	AACCC	37	n.332_333insACCC	p.Asn111_Arg112fs	groEL', 60 KDA chaperonin (protein CPN60) (HSP60)-N-terminal fragment
610992	cg0691	MISSENSE	C	A	38	n.333C>A	p.Asn111Lys	groEL', 60 KDA chaperonin (protein CPN60) (HSP60)-N-terminal fragment
610993	cg0691	MISSENSE	C	T	42	n.334C>T	p.Arg112Trp	groEL', 60 KDA chaperonin (protein CPN60) (HSP60)-N-terminal fragment
610993	cg0691	Insertion	C	CCAAT	47	n.335_336insCAAT	p.Arg112_Pro113fs	groEL', 60 KDA chaperonin (protein CPN60) (HSP60)-N-terminal fragment
610996	cg0691	MISSENSE	C	A	49	n.337C>A	p.Pro113Thr	groEL', 60 KDA chaperonin (protein CPN60) (HSP60)-N-terminal fragment
610997	cg0691	MISSENSE	C	G	42	n.338C>G	p.Pro113Arg	groEL', 60 KDA chaperonin (protein CPN60) (HSP60)-N-terminal fragment
610998	cg0691	SILENT	C	G	6	n.339C>G	p.Pro113Pro	groEL', 60 KDA chaperonin (protein CPN60) (HSP60)-N-terminal fragment
611000	cg0691	MISSENSE	T	A	38	n.341T>A	p.Phe114Tyr	groEL', 60 KDA chaperonin (protein CPN60) (HSP60)-N-terminal fragment
611001	cg0691	MISSENSE	C	A	39	n.342C>A	p.Phe114Leu	groEL', 60 KDA chaperonin (protein CPN60) (HSP60)-N-terminal fragment
611002	cg0691	MISSENSE	C	A	8	n.343C>G	p.Arg115Gly	groEL', 60 KDA chaperonin (protein CPN60) (HSP60)-N-terminal fragment
611006	cg0691	MISSENSE	T	A	10	n.347T>A	p.Phe116Tyr	groEL', 60 KDA chaperonin (protein CPN60) (HSP60)-N-terminal fragment
611009	cg0691	MISSENSE	G	C	15	n.350G>C	p.Trp117Ser	groEL', 60 KDA chaperonin (protein CPN60) (HSP60)-N-terminal fragment
611010	cg0691	NONSENSE	G	A	7	n.351G>A	p.Trp117*	groEL', 60 KDA chaperonin (protein CPN60) (HSP60)-N-terminal fragment
611012	cg0691	MISSENSE	G	C	15	n.353G>C	p.Gly118Ala	groEL', 60 KDA chaperonin (protein CPN60) (HSP60)-N-terminal fragment
611014	cg0691	MISSENSE	A	G	14	n.355A>G	p.Thr119Ala	groEL', 60 KDA chaperonin (protein CPN60) (HSP60)-N-terminal fragment
611017	cg0691	MISSENSE	T	G	12	n.358T>G	p.Ser120Ala	groEL', 60 KDA chaperonin (protein CPN60) (HSP60)-N-terminal fragment
611018	cg0691	NONSENSE	C	A	9	n.359C>A	p.Ser120*	groEL', 60 KDA chaperonin (protein CPN60) (HSP60)-N-terminal fragment
611020	cg0691	MISSENSE	C	A	9	n.361C>A	p.Gln121Lys	groEL', 60 KDA chaperonin (protein CPN60) (HSP60)-N-terminal fragment
611024	cg0691	MISSENSE	A	C	6	n.365A>C	p.Asn122Thr	groEL', 60 KDA chaperonin (protein CPN60) (HSP60)-N-terminal fragment
612436		SNV	C	G	12			
612437		SNV	A	T	11			
612438		SNV	G	T	28			
612440		SNV	A	C	23			
612441		SNV	A	C	12			
612443		Insertion	A	AAG	10			
612445		SNV	C	G	15			
612445		Insertion	C	CAACGTT	15			
612446		SNV	C	G	40			
612446		Insertion	C	CTGCGCA	25			
613757		Insertion	T	TG	359			
669584		Deletion	AGTTTCTGC	A	262			
669604		SNV	G	A	38			
844090	cg0911	SILENT	A	G	253	n.562T>C	p.Leu188Leu	inositol monophosphatase
844495	cg0911	MISSENSE	C	G	343	n.157G>C	p.Gly53Arg	inositol monophosphatase
848930		Insertion	A	AG	266			
848950		Insertion	T	TG	282			
851859		Insertion	C	CG	322			
870566	cg0938	MISSENSE	C	G	297	n.158C>G	p.Ala53Gly	cold shock protein
870568	cg0938	MISSENSE	G	C	309	n.160G>C	p.Gly54Arg	cold shock protein
892384	cg0957	MISSENSE	C	G	327	n.5242C>G	p.Pro1748Ala	fas-IB, fatty acid synthase
924555		Insertion	G	GT	242			
934508		Insertion	C	CT	262			
960472		Deletion	AG	A	306			
978319	cg1052	MISSENSE	A	C	291	n.564A>C	p.Lys188Asn	cmt3, corynomycolyl transferase
1035302		SNV	C	G	245			
1060069		Insertion	G	GA	347			
1075381	cg1159	MISSENSE	C	G	282	n.1330G>C	p.Val444Leu	putative secreted protein
1095361	cg1180	NONSENSE	CT	C	304	n.667delT	p.Phe223fs	glycosyltransferase, probably involved in cell wall biogenesis
1111143	cg1206	Insertion	T	TG	266	n.682_683insG	p.Ala228_Cys229fs	PEP phosphonomutase or related enzyme
1124440		Insertion	A	AC	277			
1126684	cg1226	MISSENSE	A	G	301	n.805T>C	p.Cys269Arg	pobB, 4-hydroxybenzoate 3-monooxygenase
1211946	cg1307	MISSENSE	A	T	311	n.545A>T	p.Gln182Leu	superfamily II DNA and RNA helicase
1232173	cg1325	SILENT	A	C	299	n.87A>C	p.Thr29Thr	putative stress-responsive transcriptional regulator
1262590	cg1351	MISSENSE	A	G	295	n.80T>C	p.Leu27Pro	moeA3, molybdopterin biosynthesis protein
1326046		Deletion	AC	A	337			
1326054		SNV	G	C	322			
1326055		SNV	C	G	321			
1326069		SNV	G	T	329			
1326070		SNV	A	G	328			
1344200		Insertion	G	GAA	318			
1396004		SNV	A	G	86			
1396005		SNV	G	T	72			
1396008		SNV	A	C	63			
1396009		SNV	C	T	61			
1396012		SNV	A	G	58			
1396014		SNV	C	G	47			
1396015		SNV	G	T	46			
1396019		SNV	C	T	44			
1396023		SNV	G	T	42			
1396025		SNV	C	T	29			
1396031		SNV	A	T	24			
1396033		SNV	C	G	24			
1396035		SNV	C	G	23			
1396036		SNV	G	T	23			
1396040		SNV	A	C	22			
1396042		SNV	A	G	21			
1396045		SNV	C	T	21			
1396112		SNV	A	T	28			
1396115		SNV	G	T	28			
1396117		SNV	A	G	28			
1396120		SNV	C	T	29			
1396131		SNV	C	T	43			
1396133		SNV	A	C	44			
1396136		SNV	G	T	67			
1396138		SNV	G	T	69			
1396139		SNV	A	G	69			
1396143		SNV	C	T	72			

1396145		SNV	A	G	73			
1396147		Insertion	G	GT	33			
1396149		SNV	C	T	73			
1396152		SNV	A	C	89			
1569394	cg1683	SILENT	T	C	265	n.1977A>G	p.Glu659Glu	superfamily II DNA and RNA helicase
1681609	cg1787	Deletion	AT	A	314	n.1delA	p.Met1fs	ppc, phosphoenolpyruvate carboxylase
1686054	cg1792	MISSENSE	A	G	266	n.512T>C	p.Ile171Thr	putative transcriptional regulator-WhiA homolog
**1691145**	**cg1796**	**MISSENSE**	**C**	**T**	**303**	**n.103G>A**	**p.Glu35Lys**	**ribX, putative membrane protein-C. ammoniagenes RibX homolog**
1714259	cg1819	MISSENSE	A	C	231	n.556A>C	p.Lys186Gln	nucleoside-diphosphate sugar epimerase (SulA family)
1716531		Deletion	TG	T	197			
1753385	cg1859	MISSENSE	C	G	335	n.420C>G	p.Asp140Glu	putative secreted protein
1753388	cg1859	SILENT	G	T	338	n.423G>T	p.Pro141Pro	putative secreted protein
1753594		Deletion	CG	C	281			
1753875	cg1860	MISSENSE	A	T	293	n.151A>T	p.Thr51Ser	hypothetical protein cg1860
1754001		Deletion	AAT	A	81			
1754014		Deletion	AAATCC	A	75			
1754021		SNV	A	T	220			
1754029		SNV	A	G	236			
1754031		SNV	C	G	14			
1754032		SNV	G	T	231			
1754033		SNV	T	C	14			
1754034		SNV	G	T	16			
1754034		Deletion	G	GGGCT	95			
1754038		Insertion	A	AC	104			
1755582	cg1861	MISSENSE	C	T	304	n.785G>A	p.Gly262Glu	rel, ppGpp synthetase, ppGpp pyrophosphorylase
1759010		SNV	T	C	277			
1763157	cg1869	MISSENSE	C	T	269	n.823G>A	p.Val275Ile	ruvB, holliday junction DNA helicase RuvB
1765670	cg1872	MISSENSE	A	T	282	n.545T>A	p.Val182Glu	hypothetical protein cg1872
1765671	cg1872	MISSENSE	C	G	283	n.544G>C	p.Val182Leu	hypothetical protein cg1872
1811091		SNV	C	T	265			
1960261	cg2066	SILENT	G	A	160	n.552C>T	p.Thr184Thr	uncharacterized low-complexity protein
1960289	cg2066	MISSENSE	T	C	201	n.524A>G	p.Asn175Ser	uncharacterized low-complexity protein
1960327	cg2066	MISSENSE	A	T	212	n.486T>A	p.His162Gln	uncharacterized low-complexity protein
1960333	cg2066	SILENT	C	T	237	n.480G>A	p.Ser160Ser	uncharacterized low-complexity protein
1960344	cg2066	MISSENSE	T	C	231	n.469A>G	p.Ser157Gly	uncharacterized low-complexity protein
1960351	cg2066	SILENT	A	T	230	n.462T>A	p.Ala154Ala	uncharacterized low-complexity protein
1960357	cg2066	SILENT	C	A	229	n.456G>T	p.Ser152Ser	uncharacterized low-complexity protein
1960366	cg2066	SILENT	G	A	240	n.447C>T	p.Cys149Cys	uncharacterized low-complexity protein
1960369	cg2066	MISSENSE	T	A	243	n.444A>T	p.Glu148Asp	uncharacterized low-complexity protein
1960379	cg2066	MISSENSE	C	T	240	n.434G>A	p.Ser145Asn	uncharacterized low-complexity protein
1960395	cg2066	MISSENSE	C	T	239	n.418G>A	p.Glu140Lys	uncharacterized low-complexity protein
1960441	cg2066	SILENT	T	C	254	n.372A>G	p.Lys124Lys	uncharacterized low-complexity protein
1960739	cg2066	MISSENSE	T	A	343	n.74A>T	p.Asp25Val	uncharacterized low-complexity protein
1961398	cg2067	MISSENSE	A	C	299	n.271T>G	p.Leu91Val	hypothetical protein cg2067
1962994	cg2069	SILENT	G	C	268	n.1485C>G	p.Val495Val	psp1, putative secreted protein
1963729	cg2069	SILENT	A	G	264	n.750T>C	p.Tyr250Tyr	psp1, putative secreted protein
1963843	cg2069	SILENT	C	T	211	n.636G>A	p.Arg212Arg	psp1, putative secreted protein
1963845	cg2069	SILENT	T	G	209	n.634A>C	p.Arg212Arg	psp1, putative secreted protein
1963857	cg2069	SILENT	G	A	212	n.622C>T	p.Leu208Leu	psp1, putative secreted protein
1963885	cg2069	SILENT	A	G	245	n.594T>C	p.His198His	psp1, putative secreted protein
1963900	cg2069	SILENT	A	T	246	n.579T>A	p.Gly193Gly	psp1, putative secreted protein
1963903	cg2069	SILENT	C	A	248	n.576G>T	p.Gly192Gly	psp1, putative secreted protein
1963908	cg2069	MISSENSE	C	A	244	n.571G>T	p.Val191Phe	psp1, putative secreted protein
1963960	cg2069	SILENT	T	C	272	n.519A>G	p.Pro173Pro	psp1, putative secreted protein
1963996	cg2069	SILENT	C	G	298	n.483G>C	p.Arg161Arg	psp1, putative secreted protein
1964002	cg2069	SILENT	A	G	295	n.477T>C	p.Ala159Ala	psp1, putative secreted protein
1964004	cg2069	MISSENSE	C	T	283	n.475G>A	p.Ala159Thr	psp1, putative secreted protein
1964014	cg2069	SILENT	C	T	279	n.465G>A	p.Arg155Arg	psp1, putative secreted protein
1964125	cg2069	MISSENSE	C	G	259	n.354G>C	p.Glu118Asp	psp1, putative secreted protein
1964134	cg2069	SILENT	C	T	295	n.345G>A	p.Val115Val	psp1, putative secreted protein
1964155	cg2069	SILENT	A	G	257	n.324T>C	p.Val108Val	psp1, putative secreted protein
1964164	cg2069	SILENT	A	G	251	n.315T>C	p.Arg105Arg	psp1, putative secreted protein
1964170	cg2069	MISSENSE	G	T	254	n.309C>A	p.Asp103Glu	psp1, putative secreted protein
1964182	cg2069	SILENT	G	T	263	n.297C>A	p.Ile99Ile	psp1, putative secreted protein
1964197	cg2069	SILENT	C	T	254	n.282G>A	p.Leu94Leu	psp1, putative secreted protein
1964203	cg2069	SILENT	A	G	225	n.276T>C	p.Leu92Leu	psp1, putative secreted protein
1964212	cg2069	SILENT	A	G	222	n.267T>C	p.Val89Val	psp1, putative secreted protein
1964218	cg2069	SILENT	G	A	218	n.261C>T	p.Asp87Asp	psp1, putative secreted protein
1964236	cg2069	MISSENSE	G	T	191	n.243C>A	p.His81Gln	psp1, putative secreted protein
1964253	cg2069	MISSENSE	T	C	181	n.226A>G	p.Ile76Val	psp1, putative secreted protein
1964274	cg2069	MISSENSE	A	T	172	n.205T>A	p.Ser69Thr	psp1, putative secreted protein
1964279	cg2069	MISSENSE	A	T	173	n.200T>A	p.Phe67Tyr	psp1, putative secreted protein
1964298	cg2069	MISSENSE	T	C	165	n.181A>G	p.Lys61Glu	psp1, putative secreted protein
1964302	cg2069	SILENT	A	T	172	n.177T>A	p.Thr59Thr	psp1, putative secreted protein
1964305	cg2069	SILENT	C	T	173	n.174G>A	p.Pro58Pro	psp1, putative secreted protein
1964329	cg2069	SILENT	G	A	195	n.150C>T	p.Asp50Asp	psp1, putative secreted protein
1964338	cg2069	SILENT	A	G	198	n.141T>C	p.Asp47Asp	psp1, putative secreted protein
1964352	cg2069	NONSENSE	G	A	214	n.127C>T	p.Gln43*	psp1, putative secreted protein
1964360	cg2069	MISSENSE	C	G	212	n.119G>C	p.Ser40Thr	psp1, putative secreted protein
1964370	cg2069	MISSENSE	T	C	221	n.109A>G	p.Lys37Glu	psp1, putative secreted protein
1964395	cg2069	SILENT	T	C	249	n.84A>G	p.Gln28Gln	psp1, putative secreted protein
1964407	cg2069	SILENT	T	C	256	n.72A>G	p.Val24Val	psp1, putative secreted protein
1964440	cg2069	SILENT	G	A	273	n.39C>T	p.Val13Val	psp1, putative secreted protein
1964452	cg2069	SILENT	A	C	270	n.27T>G	p.Gly9Gly	psp1, putative secreted protein
1964477	cg2069	MISSENSE	A	T	135	n.2T>A	p.Met1?	psp1, putative secreted protein
1964507		SNV	G	A	91			
1964515		SNV	G	A	85			
1964525		SNV	A	G	76			
1964527		SNV	C	T	74			
1964529		SNV	T	A	69			
1964929	cg2071	SILENT	T	C	186	n.414A>G	p.Ala138Ala	int2', putative phage integrase (N-terminal fragment)
1964947	cg2071	SILENT	C	A	194	n.396G>T	p.Gly132Gly	int2', putative phage integrase (N-terminal fragment)
1964950	cg2071	SILENT	A	G	208	n.393T>C	p.Asp131Asp	int2', putative phage integrase (N-terminal fragment)
1964959	cg2071	SILENT	G	A	218	n.384C>T	p.Thr128Thr	int2', putative phage integrase (N-terminal fragment)
1964962	cg2071	SILENT	G	A	219	n.381C>T	p.Asp127Asp	int2', putative phage integrase (N-terminal fragment)
1964992	cg2071	SILENT	G	A	252	n.351C>T	p.Thr117Thr	int2', putative phage integrase (N-terminal fragment)
1964999	cg2071	MISSENSE	G	T	270	n.344C>A	p.Ala115Glu	int2', putative phage integrase (N-terminal fragment)
1965001	cg2071	SILENT	G	A	270	n.342C>T	p.Cys114Cys	int2', putative phage integrase (N-terminal fragment)
1965079	cg2071	SILENT	A	G	328	n.264T>C	p.Leu88Leu	int2', putative phage integrase (N-terminal fragment)
1965133	cg2071	SILENT	A	G	325	n.210T>C	p.Phe70Phe	int2', putative phage integrase (N-terminal fragment)
1965160	cg2071	SILENT	A	G	313	n.183T>C	p.Ala61Ala	int2', putative phage integrase (N-terminal fragment)
1965163	cg2071	SILENT	C	T	306	n.180G>A	p.Glu60Glu	int2', putative phage integrase (N-terminal fragment)
1965166	cg2071	SILENT	A	G	308	n.177T>C	p.Leu59Leu	int2', putative phage integrase (N-terminal fragment)
1965167	cg2071	MISSENSE	A	C	310	n.176T>G	p.Leu59Arg	int2', putative phage integrase (N-terminal fragment)
1965176	cg2071	MISSENSE	C	T	324	n.167G>A	p.Arg56Gln	int2', putative phage integrase (N-terminal fragment)
1965181	cg2071	SILENT	A	G	307	n.162T>C	p.Phe54Phe	int2', putative phage integrase (N-terminal fragment)
1965229	cg2071	SILENT	T	C	254	n.114A>G	p.Thr38Thr	int2', putative phage integrase (N-terminal fragment)
1965253	cg2071	MISSENSE	A	C	251	n.90T>G	p.Asn30Lys	int2', putative phage integrase (N-terminal fragment)
1965255	cg2071	MISSENSE	T	G	254	n.88A>C	p.Asn30His	int2', putative phage integrase (N-terminal fragment)
1965256	cg2071	SILENT	A	C	250	n.87T>G	p.Ala29Ala	int2', putative phage integrase (N-terminal fragment)
1965277	cg2071	SILENT	A	G	229	n.66T>C	p.Thr22Thr	int2', putative phage integrase (N-terminal fragment)
1965280	cg2071	SILENT	G	A	236	n.63C>T	p.Tyr21Tyr	int2', putative phage integrase (N-terminal fragment)
1965332	cg2071	MISSENSE	T	C	246	n.11A>G	p.Glu4Gly	int2', putative phage integrase (N-terminal fragment)
1965346		SNV	T	C	240			#N/A
1965358		SNV	T	C	262			#N/A
1965399		SNV	T	C	283			#N/A

1965462		SNV	A	G	297			#N/A
1965468		SNV	C	T	297			#N/A
1965491		SNV	T	C	297			#N/A
1965543		SNV	G	A	281			#N/A
1965560		SNV	A	G	260			#N/A
1965581		SNV	C	T	222			#N/A
1965584		SNV	T	C	225			#N/A
1965588		SNV	G	T	217			#N/A
1965603		SNV	A	G	184			#N/A
1965633		SNV	T	C	131			#N/A
1965646		SNV	C	T	108			#N/A
1965652		SNV	A	G	108			#N/A
1965681		SNV	A	G	87			#N/A
1965683		SNV	G	A	87			#N/A
1995296	cg2105	MISSENSE	G	A	311	n.23C>T	p.Pro8Leu	hypothetical protein cg2105
2002299	cg2111	SILENT	G	T	263	n.774G>T	p.Arg258Arg	hrpA, probable ATP-dependent RNA helicase protein
2005931		Deletion	AC	A	241			#N/A
2006773		SNV	A	C	287			#N/A
**2012000**	**cg2118**	**MISSENSE**	**G**	**T**	**270**	**n.224G>T**	**p.Gly75Val**	**transcriptional regulator of sugar metabolism, DeoR family**
**2028992**	**cg2135**	**MISSENSE**	**A**	**G**	**253**	**n.490T>C**	**p.Ser164Pro**	**miaB, trna methylthiotransferase**
2030556		SNV	C	T	274			#N/A
2043117		Deletion	GA	G	225			#N/A
2055662	cg2167	MISSENSE	C	G	272	n.91G>C	p.Val31Leu	rpsO, 30S ribosomal protein S15
2066532	cg2176	MISSENSE	C	G	312	n.242G>C	p.Arg81Pro	infB, translation initiation factor IF-2
2069867	cg2180	NONSENSE	T	A	244	n.818T>A	p.Leu273*	putative secreted protein
2069868	cg2180	SILENT	A	G	244	n.819A>G	p.Leu273Leu	putative secreted protein
2075725	cg2184	MISSENSE	T	C	19	n.1703T>C	p.Ile568Thr	ATPase component of peptide ABC-type transport system, contains duplicated ATPase domains
2075727	cg2184	MISSENSE	G	C	25	n.1705G>C	p.Glu569Gln	ATPase component of peptide ABC-type transport system, contains duplicated ATPase domains
2075732	cg2184	MISSENSE	G	C	30	n.1710G>C	p.Leu570Phe	ATPase component of peptide ABC-type transport system, contains duplicated ATPase domains
2075733	cg2184	MISSENSE	G	T	30	n.1711G>T	p.Gly571Cys	ATPase component of peptide ABC-type transport system, contains duplicated ATPase domains
2075736	cg2184	MISSENSE	A	G	32	n.1714A>G	p.Thr572Ala	ATPase component of peptide ABC-type transport system, contains duplicated ATPase domains
2075737	cg2184	MISSENSE	C	A	32	n.1715C>A	p.Thr572Asn	ATPase component of peptide ABC-type transport system, contains duplicated ATPase domains
2075740	cg2184	MISSENSE	G	T	35	n.1718G>T	p.Gly573Val	ATPase component of peptide ABC-type transport system, contains duplicated ATPase domains
2075742	cg2184	MISSENSE	G	C	52	n.1720G>C	p.Glu574Gln	ATPase component of peptide ABC-type transport system, contains duplicated ATPase domains
2075747	cg2184	Insertion	C	CCGGAAC	21	n.1726_1727insCGGAAGG	p.Leu576_Val577fs	ATPase component of peptide ABC-type transport system, contains duplicated ATPase domains
2075749	cg2184	MISSENSE	T	C	60	n.1727T>C	p.Leu576Pro	ATPase component of peptide ABC-type transport system, contains duplicated ATPase domains
2075759		SNV	C	G	28			
2075762		SNV	A	T	27			
2075764		SNV	A	T	23			
2075765		SNV	G	T	23			
2130787		SNV	C	T	256			
2144955	cg2262	SILENT	A	T	43	n.582T>A	p.Val194Val	ftsY, signal recognition particle GTPase
2144956	cg2262	MISSENSE	A	G	43	n.581T>C	p.Val194Ala	ftsY, signal recognition particle GTPase
2144958	cg2262	SILENT	A	T	47	n.579T>A	p.Ile193Ile	ftsY, signal recognition particle GTPase
2144960	cg2262	MISSENSE	T	C	52	n.577A>G	p.Ile193Val	ftsY, signal recognition particle GTPase
2144964	cg2262	SILENT	T	A	59	n.573A>T	p.Pro191Pro	ftsY, signal recognition particle GTPase
2144964	cg2262	Insertion	T	TGGTTCC	138	n.572_573insTGCAGTAGC	p.Pro191_Ala192insProAlaValAlaGluGluPro	ftsY, signal recognition particle GTPase
2145009	cg2262	SILENT	A	T	47	n.528T>A	p.Val176Val	ftsY, signal recognition particle GTPase
2145010	cg2262	MISSENSE	A	G	45	n.527T>C	p.Val176Ala	ftsY, signal recognition particle GTPase
2145012	cg2262	SILENT	A	T	43	n.525T>A	p.Ile175Ile	ftsY, signal recognition particle GTPase
2145014	cg2262	MISSENSE	T	C	38	n.523A>G	p.Ile175Val	ftsY, signal recognition particle GTPase
2145018	cg2262	SILENT	T	A	34	n.519A>T	p.Pro173Pro	ftsY, signal recognition particle GTPase
2145028	cg2262	MISSENSE	G	A	25	n.509C>T	p.Ala170Val	ftsY, signal recognition particle GTPase
2145398	cg2262	MISSENSE	G	T	302	n.139C>A	p.Pro47Thr	ftsY, signal recognition particle GTPase
2166304		Insertion	A	AC	330			
2212356	cg2331	MISSENSE	A	T	310	n.36T>A	p.Asp12Glu	hypothetical protein cg2331
2218242	cg2337	Deletion	GA	G	321	n.10delT	p.Ser4fs	similar to methionine synthase II (cobalamin-independent)
2236682	cg2353	Insertion	C	CGAATTA	22	n.229_230insGAATTA	p.Gly77_Arg78insGluLeuGly	hypothetical protein disrupted by insertion of ISCg2e
2236687	cg2353	MISSENSE	G	A	35	n.233G>A	p.Arg78Gln	hypothetical protein disrupted by insertion of ISCg2e
2236693	cg2354	MISSENSE	A	G	38	n.1513T>C	p.Ter505Argext*?	tnp2e(ISCg2e), transposase
2236697	cg2353	SILENT	G	A	35	n.243G>A	p.Ser81Ser	hypothetical protein disrupted by insertion of ISCg2e
2236702	cg2353	MISSENSE	A	C	24	n.248A>C	p.Asp83Ala	hypothetical protein disrupted by insertion of ISCg2e
2236703	cg2353	SILENT	T	C	29	n.249T>C	p.Asp83Asp	hypothetical protein disrupted by insertion of ISCg2e
2238303		SNV	C	A	8			
2238310		Insertion	T	TTAAATC	12			
2238314		SNV	C	T	14			
2238314		Insertion	C	CGAATTT	14			
2238317		SNV	G	C	59			
2245932		SNV	T	C	314			
2245934		SNV	C	A	312			
2253306	cg2368	SILENT	A	G	251	n.990T>C	p.Phe330Phe	murC, UDP-N-acetylmuramate--L-alanine ligase
2267578		Deletion	AC	A	234			
**2268301**	**cg2380**	**Deletion**	**GC**	**G**	**262**	**n.270delG**	**p.Gly90fs**	**hypothetical protein cg2380**
2279307		Insertion	T	TG	142			
**2302995**	**cg2412**	**MISSENSE**	**C**	**T**	**298**	**n.154C>T**	**p.Pro52Ser**	**hypothetical protein cg2412**
2327189		Deletion	CT	C	254			
2335862		Deletion	TG	T	269			
2339424		Insertion	G	GT	282			
2345684	cg2457	Deletion	AG	A	234	n.451delC	p.Leu151fs	hypothetical protein cg2457
2345701	cg2457	Insertion	T	TG	233	n.434_435insC	p.Pro145_Arg146fs	hypothetical protein cg2457
2347748	cg2460	SILENT	A	G	291	n.354A>G	p.Glu118Glu	hypothetical protein cg2460
2348658		SNV	T	C	42			
2348667		SNV	G	A	85			
2348740	cg2461	SILENT	G	A	203	n.324C>T	p.Ile108Ile	tnp4a(ISCg4a), transposase
2348776	cg2461	SILENT	G	A	226	n.288C>T	p.Val96Val	tnp4a(ISCg4a), transposase
2348788	cg2461	SILENT	T	A	222	n.276A>T	p.Arg92Arg	tnp4a(ISCg4a), transposase
2348862	cg2461	MISSENSE	C	T	209	n.202G>A	p.Ala68Thr	tnp4a(ISCg4a), transposase
2348880	cg2461	MISSENSE	T	C	199	n.184A>G	p.Lys62Glu	tnp4a(ISCg4a), transposase
2348888	cg2461	MISSENSE	G	A	226	n.176C>T	p.Thr59Ile	tnp4a(ISCg4a), transposase
2348912	cg2461	MISSENSE	C	A	185	n.152G>T	p.Arg51Leu	tnp4a(ISCg4a), transposase
2348913	cg2461	MISSENSE	G	A	184	n.151C>T	p.Arg51Cys	tnp4a(ISCg4a), transposase
2348987	cg2461	MISSENSE	C	G	132	n.77G>C	p.Cys26Ser	tnp4a(ISCg4a), transposase
2348989	cg2461	SILENT	A	G	131	n.75T>C	p.Gly25Gly	tnp4a(ISCg4a), transposase
2348992	cg2461	SILENT	T	C	129	n.72A>G	p.Gly24Gly	tnp4a(ISCg4a), transposase
2349035	cg2461	MISSENSE	G	A	88	n.29C>T	p.Ser10Leu	tnp4a(ISCg4a), transposase
2349156		SNV	A	G	130			
2349159		SNV	T	A	131			
2349197		SNV	G	T	152			
2349213		SNV	T	G	156			
2349224		SNV	A	G	154			
2349242		SNV	G	A	183			
2349276		SNV	A	C	194			
2349300		SNV	G	C	196			
2349326		SNV	T	A	192			
2349387		SNV	A	G	146			
2350220		SNV	T	G	305			
2354473		SNV	C	G	294			
2354476		SNV	C	T	289			
2354634	cg2467	MISSENSE	C	G	294	n.655G>C	p.Val219Leu	ABC transporter ATP-binding protein
2359858	cg2474	MISSENSE	A	T	304	n.189A>T	p.Glu63Asp	nagD, putative phosphatase in N-acetylglucosamine metabolism
2361038	cg2475	MISSENSE	A	C	282	n.528A>C	p.Lys176Asn	ATPase component of ABC transporters with duplicated ATPase domains
2361044	cg2475	SILENT	A	C	284	n.534A>C	p.Arg178Arg	ATPase component of ABC transporters with duplicated ATPase domains
2361919		Deletion	CT	C	270			
2361924		Deletion	CT	C	265			
2386709	cg2504	SILENT	A	G	183	n.1467A>G	p.Lys489Lys	hypothetical protein cg2504
2386727	cg2504	SILENT	C	G	153	n.1485C>G	p.Pro495Pro	hypothetical protein cg2504

2397447	cg2517	SILENT	G	A	790	n.333C>T	p.Phe111Phe	hemN, coproporphyrinogen III oxidase
2429490		Deletion	TA	T	943			
2441887		Deletion	GT	G	928			
2475063		Insertion	A	AC	775			
**2517405**	**cg2637**	**MISSENSE**	**G**	**A**	**1001**	**n.436G>A**	**p.Asp146Asn**	**benA, benzoate 1,2-dioxygenase alpha subunit (aromatic ring hydroxylation dioxygenase A)**
2528305		SNV	A	C	101			
2528309		SNV	T	A	88			
2528317		SNV	T	A	214			
2528319		SNV	T	G	221			
2528320		SNV	T	G	330			
2528321		SNV	T	G	273			
2528333		SNV	C	G	278			
2528340		SNV	T	C	200			
2528342		SNV	G	T	188			
2528343		SNV	C	G	178			
2528345		SNV	A	T	230			
2528347		SNV	C	T	180			
2528350		SNV	C	A	162			
2528353		SNV	T	A	138			
2528356		SNV	C	G	147			
2528358		SNV	C	T	103			
2558065	cg2683	SILENT	A	C	255	n.177A>C	p.Thr59Thr	hypothetical protein cg2683
2558149	cg2683	SILENT	C	T	496	n.261C>T	p.Ser87Ser	hypothetical protein cg2683
2558213	cg2683	MISSENSE	C	T	288	n.325C>T	p.Pro109Ser	hypothetical protein cg2683
2575134		Insertion	A	AC	363			
2575624	cg2701	MISSENSE	A	G	247	n.635T>C	p.Phe212Ser	hypothetical protein cg2701
**2579158**	**cg2705**	**MISSENSE**	**G**	**A**	**297**	**n.407C>T**	**p.Ala136Val**	**amyE, maltose-binding protein precursor**
2584856	cg2712	Insertion	G	GC	287	n.454_455insG	p.Ala152_Cys153fs	AraC-type regulator
2675616		SNV	G	A	109			
2675617		Insertion	T	TA	6			
2680620	cgr12	Insertion	C	CA	31	n.1_2insT	p.Cys1_Gly2fs	16S ribosomal RNA
2714348		Insertion	T	TC	296			
2716175		Insertion	G	GC	165			
2716279		Insertion	C	CT	66			
2716281		SNV	G	T	20			
2716282		SNV	C	A	65			
2716283		SNV	G	T	44			
2716284		SNV	A	T	41			
2716285		SNV	A	T	19			
2716287	cg2854	MISSENSE	T	G	41	n.1515A>C	p.Ter505Cysext*?	tnp2c(ISCg2c), transposase
2716293	cg2854	SILENT	G	C	38	n.1509C>G	p.Thr503Thr	tnp2c(ISCg2c), transposase
2716298	cg2854	MISSENSE	A	C	25	n.1504T>G	p.Ser502Ala	tnp2c(ISCg2c), transposase
2716300	cg2854	MISSENSE	G	T	27	n.1502C>A	p.Ala501Glu	tnp2c(ISCg2c), transposase
2717903		SNV	C	G	11			
2717911		SNV	C	G	34			
2717912		SNV	C	A	22			
2717913		SNV	G	A	22			
2717915		SNV	C	T	46			
2736757		Deletion	AT	A	226			
**2799568**	**cg2941**	**MISSENSE**	**T**	**C**	**301**	**n.577A>G**	**p.Ile193Val**	**lyse type translocator**
2816684		Insertion	A	AG	337			
2855585	cg3003	MISSENSE	G	A	379	n.1280G>A	p.Arg427Gln	cps, non-ribosomal peptide synthetase
2877096	cg3018	MISSENSE	A	G	306	n.133T>C	p.Ser45Pro	hypothetical protein cg3018
2913128		Insertion	G	GC	273			
2913143		Insertion	A	AT	279			
2920095	cg3058	MISSENSE	C	T	146	n.803G>A	p.Arg268Lys	tnp8b(ISCg8a), transposase
2920096	cg3058	MISSENSE	T	A	156	n.802A>T	p.Arg268Trp	tnp8b(ISCg8a), transposase
2920106	cg3058	SILENT	G	C	173	n.792C>G	p.Arg264Arg	tnp8b(ISCg8a), transposase
2920121	cg3058	SILENT	G	A	187	n.777C>T	p.Arg259Arg	tnp8b(ISCg8a), transposase
2920139	cg3058	SILENT	G	A	208	n.759C>T	p.Ala253Ala	tnp8b(ISCg8a), transposase
2920166	cg3058	SILENT	C	T	230	n.732G>A	p.Glu244Glu	tnp8b(ISCg8a), transposase
2920169	cg3058	SILENT	T	C	235	n.729A>G	p.Arg243Arg	tnp8b(ISCg8a), transposase
2920178	cg3058	SILENT	G	C	235	n.720C>G	p.Ala240Ala	tnp8b(ISCg8a), transposase
2920199	cg3058	SILENT	C	T	258	n.699G>A	p.Leu233Leu	tnp8b(ISCg8a), transposase
2920313	cg3058	SILENT	C	A	161	n.585G>T	p.Val195Val	tnp8b(ISCg8a), transposase
2920316	cg3058	SILENT	C	A	160	n.582G>T	p.Ala194Ala	tnp8b(ISCg8a), transposase
2920324	cg3058	MISSENSE	T	G	166	n.574A>C	p.Lys192Gln	tnp8b(ISCg8a), transposase
2920325	cg3058	SILENT	C	T	166	n.573G>A	p.Leu191Leu	tnp8b(ISCg8a), transposase
2920328	cg3058	SILENT	G	A	158	n.570C>T	p.Ser190Ser	tnp8b(ISCg8a), transposase
2920354	cg3058	MISSENSE	G	T	120	n.544C>A	p.Leu182Met	tnp8b(ISCg8a), transposase
2920358	cg3058	SILENT	T	C	120	n.540A>G	p.Ala180Ala	tnp8b(ISCg8a), transposase
2921127	cg3059	SILENT	G	A	128	n.66C>T	p.Asp22Asp	tnp8a(ISCg8a), transposase
2921175	cg3059	SILENT	A	G	138	n.18T>C	p.Tyr6Tyr	tnp8a(ISCg8a), transposase
2921196		SNV	C	A	108			
2921213		SNV	A	G	73			
3028090	cg3170	MISSENSE	A	G	269	n.32A>G	p.Asn11Ser	tellurite resistance protein or related permease
3046767	cg3182	MISSENSE	C	T	330	n.160G>A	p.Val54Ile	cop1, trehalose corynomycolyl transferase
3051028	cg3187	MISSENSE	C	G	301	n.1051G>C	p.Val351Leu	aftB, arabinofuranosyltransferase
3051152	cg3187	SILENT	C	T	273	n.927G>A	p.Glu309Glu	aftB, arabinofuranosyltransferase
3061629		Deletion	AT	A	377			
3185192		Deletion	GA	G	298			
3186651	cg3339	MISSENSE	A	C	248	n.756A>C	p.Glu252Asp	merA, putative FAD-dependent pyridine nucleotide-disulphideoxidoreductase, similar to mercuric reductases
3219803	cg3370	MISSENSE	C	G	313	n.410G>C	p.Gly137Ala	putative NADH-dependent flavin oxidoreductase
3282705		SNV	C	T	18			
3282707		SNV	T	G	19			
3282708		SNV	A	C	9			

Note: Reference Position: Variant position on reference genome; Type: Variant type. SNV (single-nucleotide variant), MNV (multi-nucleotide variant), InDel(insertion or deletion); Reference: Reference nucleotide (ATGC) sequence at the position of the variant; Allele: The alleles found by the variant caller in the sequencing data; Counts: The number of reads supporting the alleles; For variants that fall within a coding region of a gene, the change is reported.;The report of synonymous or non-synonymous changes

Highlights: Common mutations of Cg-Cello1(evo) and CgCello2(evo)

Table S3. List of genes with significantly changed gene expression in the Group (2) and Group (3)

Name	Cg-Cello01(evo)-glucose/ Cg-pbEbEB1c-glucose	Cg-Cello01(evo)-cellobiose/ Cg-pbEbEB1c-glucose[a]	Cg-Cello01(evo)-cellobiose/ Cg-Cello01(evo)-glucose[a]	Gene Product
cg0104	1.730	5.005	2.893	CREATININE DEAMINASE
cg1567[b]	0.906	3.938	4.348	hypothetical protein predicted by
cg0867	1.901	3.926	2.065	Ribosome-associated protein Y (PSrp-1)
cg3420	0.515	3.699	7.184	RNA POLYMERASE SIGMA-70 FACTOR, ECF SUBFAMILY
**cg3020[b]**	**0.941**	**3.601**	**3.826**	**putative membrane protein**
**cg2380**	**1.482**	**3.570**	**2.408**	**putative membrane protein**
cg1917	1.620	3.361	2.074	hypothetical protein predicted by
cg1346	1.392	3.270	2.348	PUTATIVE MOLYBDOPTERIN BIOSYNTHESIS MOG PROTEIN
cg1968	1.107	3.266	2.950	hypothetical protein predicted by
cg2960	1.558	3.181	2.042	hypothetical protein predicted by
cg3327	1.384	2.968	2.144	STARVATION-INDUCED DNA PROTECTING PROTEIN
cg1716	0.803	2.958	3.686	transposase
cg2381[b]	1.197	2.909	2.429	conserved hypothetical protein
cg2193	0.736	2.837	3.852	putative lysophospholipase
cg2999	1.298	2.751	2.120	PUTATIVE FERREDOXIN REDUCTASE
**cg1758**	**1.078**	**2.744**	**2.546**	**putative membrane protein**
cg3328	1.054	2.730	2.591	PROBABLE FORMAMIDOPYRIMIDINE-DNA GLYCOSYLASE
cg2026	1.333	2.700	2.026	hypothetical protein predicted by Glimmer
cg3019	0.816	2.680	3.286	putative secreted protein
cg1179	0.967	2.623	2.711	SENSORY BOX/GGDEF FAMILY PROTEIN
cg2241	1.155	2.522	2.183	PUTATIVE TRANSCRIPTIONAL ACCESSORY PROTEIN, RNA
cg3082	1.030	2.446	2.373	Bacterial regulatory proteins, ArsR family
cg1476	0.989	2.357	2.383	THIAMINE BIOSYNTHESIS PROTEIN
**cg1919**	**0.736**	**2.126**	**2.889**	**putative membrane protein**
cg1244	0.902	2.085	2.310	Arsenate reductase or related protein,
cg1174	0.848	2.078	2.451	PROBABLE ORNITHINE CARBAMOYLTRANSFERASE PROTEIN
cg1308	0.713	2.015	2.825	Bacterial regulatory proteins, tetR family
cg3410	0.801	0.396	0.494	conserved hypothetical protein
cg2637	0.989	0.360	0.364	BENZOATE 1,2-DIOXYGENASE ALPHA SUBUNIT (AROMATIC
cg0040	1.977	0.357	0.181	PUTATIVE SECRETED PROTEIN
cg2465	1.551	0.115	0.074	hypothetical protein
cg2493	0.510	0.095	0.187	hypothetical protein

Name	Cg-Cello02(evo)-glucose/ Cg-pbEbEB1c-glucose	Cg-Cello02(evo)-cellobiose/ Cg-pbEbEB1c-glucose[a]	Cg-Cello02(evo)-cellobiose/ Cg-Cello02(evo)-glucose[a]	Gene Product
cg0226	1.336	7.122	5.332	TRANSPOSASE
cg2715	1.088	4.802	4.413	conserved hypothetical protein
**cg2699**	**1.707**	**4.715**	**2.762**	**putative membrane protein**
cg2950	1.408	4.636	3.293	PROBABLE ATP-DEPENDENT PROTEASE, DNA REPAIR
cg0951	0.923	4.237	4.592	ACETYL-COENZYME A CARBOXYLASE CARBOXYL
cg3248	1.653	4.031	2.438	probable two component sensor kinase
cg1665	1.388	3.940	2.840	putative secreted protein
cg1211	1.129	3.802	3.368	PUTATIVE MARR-FAMILY TRANSCRIPTIONAL REGULATOR
cg2381[b]	1.420	3.745	2.638	conserved hypothetical protein
**cg0468**	**1.206**	**3.337**	**2.766**	**cobalamin/Fe3+-siderophores transport systems,**
cg1567[b]	1.046	3.312	3.166	hypothetical protein predicted by
cg3264	1.376	3.276	2.381	conserved hypothetical protein
cg1831	0.860	3.273	3.806	Bacterial regulatory protein, arsR family
cg2776	1.551	3.243	2.091	PROBABLE ATP-DEPENDENT DNA HELICASE-RELATED
**cg3020[b]**	**1.158**	**3.181**	**2.746**	**putative membrane protein**
cg2794	1.452	3.168	2.182	conserved hypothetical protein
cg0958	0.902	3.155	3.496	secreted protein
cg2720	1.483	3.110	2.097	secreted lipoprotein ErfK/YbiS/YcfS/YnhG family
cg1944	1.247	3.077	2.468	hypothetical protein predicted by
cg1098	0.915	2.960	3.234	Bacterial regulatory proteins, tetR family
**cg1719**	**1.101**	**2.887**	**2.622**	**ABC-type multidrug/protein/lipid transport**
cg0533	0.527	2.860	5.427	O-SUCCINYLBENZOIC ACID-COA LIGASE
cg1799	1.246	2.750	2.207	PUTATIVE RIBOFLAVIN SYNTHASE
cg0662	0.792	2.713	3.424	FAD/FMN-containing dehydrogenase
cg1292	1.177	2.691	2.286	FLAVIN-CONTAINING MONOOXYGENASE 3
cg2793	1.112	2.658	2.389	conserved hypothetical protein
cg0954	1.232	2.619	2.126	putative secreted protein
cg0410	1.193	2.610	2.187	PUTATIVE PROLYL ENDOPEPTIDASE
**cg2425**	**1.253**	**2.606**	**2.079**	**Predicted permease**
cg0570	0.679	2.597	3.825	PUTATIVE DEHYDROGENASE
cg2118	0.514	2.582	5.025	transcriptional regulator of sugar metabolism,
cg1320	0.724	2.529	3.494	LIPASE
**cg0216**	**0.971**	**2.505**	**2.581**	**hypothetical membrane protein**
cg2187	1.069	2.501	2.340	Mg-chelatase subunit D
**cg3267**	**0.878**	**2.478**	**2.822**	**putative membrane protein**
**cg1289**	**1.053**	**2.470**	**2.347**	**Permease of the major facilitator superfamily**
**cg2688**	**0.700**	**2.446**	**3.494**	**ABC-type molybdenum transport system, ATPase**
cg1311	0.972	2.431	2.500	CATECHOL 1,2-DIOXYGENASE
cg0188	0.535	2.414	4.513	conserved hypothetical protein
cg0077	1.168	2.413	2.067	Conserved HYPOTHETICAL PROTEIN
cg1191	0.516	2.406	4.666	hypothetical protein predicted by
**cg2870**	**0.793**	**2.400**	**3.029**	**Na+/H+-dicarboxylate symporter**
cg0051	0.921	2.390	2.594	PROBABLE TRANSCRIPTION REGULATOR PROTEIN, AraC
cg3345	0.842	2.367	2.810	hypothetical protein predicted by
cg0073	0.650	2.365	3.638	sulfurtransferase
cg2554	1.127	2.348	2.084	PROBABLE RIBOKINASE PROTEIN
cg1804	1.014	2.338	2.305	POLYPEPTIDE DEFORMYLASE
**cg0049**	**0.597**	**2.323**	**3.889**	**PUTATIVE TRANSMEMBRANE PROTEIN, RHOMBOID FAMILY**

cg0823	0.892	2.321	2.603	NITRILOTRIACETATE MONOOXYGENASE COMPONENT A
cg3243	0.811	2.288	2.821	Predicted RecB family nuclease
cg2141	1.109	2.271	2.048	DNA RECOMBINATION/REPAIR
cg1471	0.717	2.269	3.165	hypothetical protein predicted by
**cg0455**	**1.022**	**2.259**	**2.211**	**permease, major facilitator superfamily**
cg3172	1.076	2.215	2.058	SAM-dependent methyltransferase
cg2730	0.621	2.203	3.547	Transcriptional regulator, C-terminus
cg0788	1.071	2.191	2.046	PHOSPHOGLUCOMUTASE/PHOSPHOMANNOMUTASE
**cg0752**	**0.845**	**2.153**	**2.549**	**PUTATIVE secreted or MEMBRANE PROTEIN**
cg0427	1.030	2.092	2.031	TRANSPOSASE-fragment
**cg2356**	**1.031**	**2.075**	**2.013**	**Permease of the drug/metabolite transporter**
cg2959	1.002	2.054	2.049	putative secreted protein
cg1350	0.779	2.022	2.596	PUTATIVE MOLYBDOPTERIN-GUANINE DINUCLEOTIDE
cg0474	0.879	0.407	0.463	conserved hypothetical protein
cg1718	0.914	0.380	0.416	Phospholipid-binding protein
cg0286	0.735	0.363	0.493	CONSERVED HYPOTHETICAL membrane PROTEIN
cg1016	0.952	0.343	0.361	glycine betaine transporter
cg0795	1.112	0.200	0.180	FAD-dependent pyridine nucleotide-disulphide

[a]The mRNA ratios are averages from at least two expriments.
Only values for open reading frames whose mRNA ratio was alterd at least 2-folds
for Cg-Cello01(evo)-glucose/Cg-pbEbEB1c-glucose & Cg-Cello02(evo)-glucose/Cg-pbEbEB1c-glucose.

[b]The genes were altered for in Cg-Cello01(evo) and Cg-Cello02(evo)

[c]The bolded genes were annoated as membrane bound proteins.

Log(2) ratio	Fold	Color	RGB
>3.3219280	>10		150.5.1
>2.9068906	>7.5		200.7.2
>2.3219280	>5		250.8.2
>1.5849625	>3		253.52.47
>1	>2		253.102.98
>0.8073549	>1.75		254.152.148
>0.5849625	>1.5		255.202.201
>0.3219281	>1.25		255.230.229
0	1		255.255.255
<-0.3219281	<0.8		232.229.255
<-0.5849625	<0.6666666		205.205.205
<-0.8073549	<0.5714286		152.162.254
<-1	<0.5		88.104.254
<-1.5849625	<0.3333333		39.59.253
<-2.3219280	<0.2		2.25.244
<-2.9068906	<0.1333333		1.14.137
<-3.3219280	<0.1		1.9.83

Fig. S1. Sequence analysis of the isolated plasmids from the *Cg-Cello01(evo)* strain

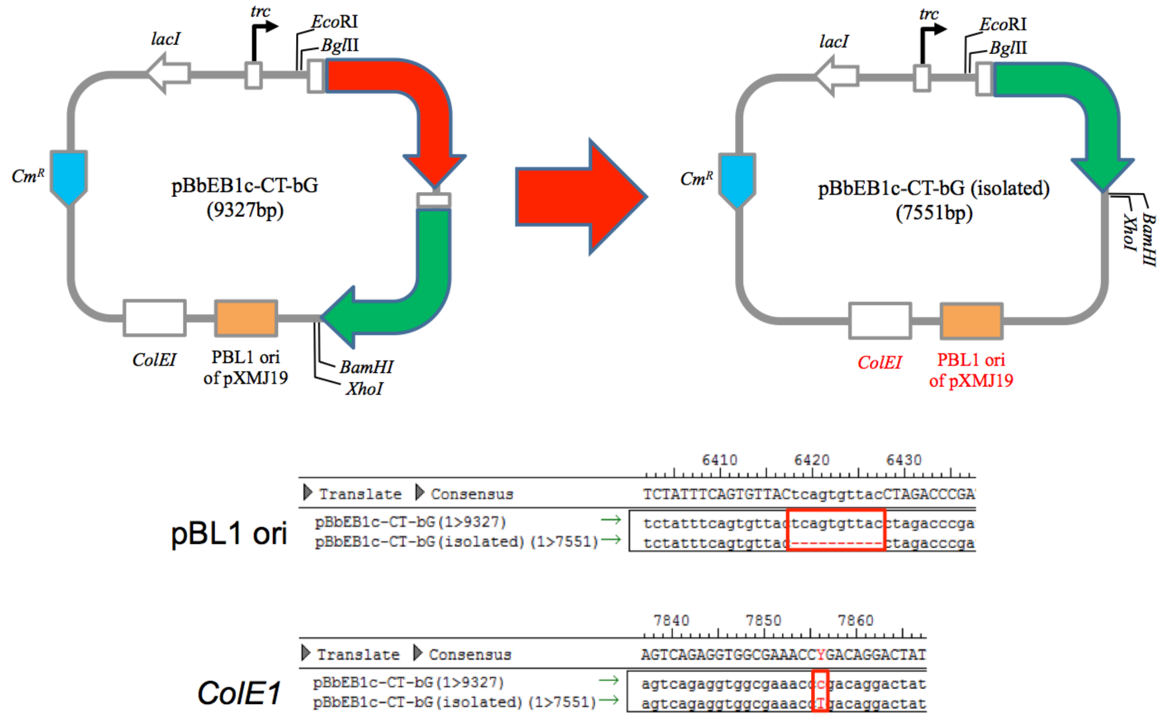

pBL ori sequence : 1376 ~ 1385 TCAGTGTTAC deletion

ColE1 sequence : 87 C->T point mutation

Fig. S2. Gel images of colony PCR products of the *cdt-1* gene on the pBbEB1c-CT-bG and scheme of intra-molecular recombination at the identical ribosomal binding site.

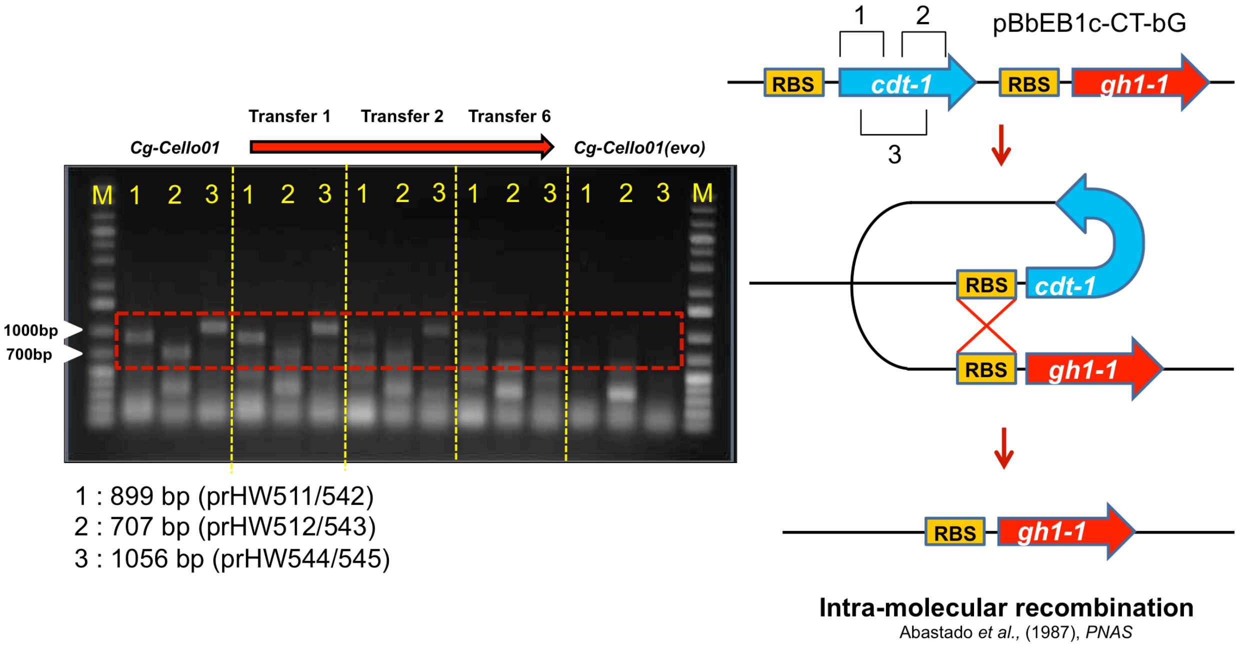

Oligo name	DNA seq. (5’ → 3’)
prHW 511-fw	atgtcctcccacggctccca
prHW 542-rv	ccgtcgcgcatttcttcggt
prHW 512-fw	accgaagaaatgcgcgacgg
prHW 543-rv	acggtttcgatcaggtcccagc
prHW 544-fw	gtcatcttctccctgtacaccgt
prHW 545-rv	gagtggggtgtaggtgaaggag

Fig. S3. Scatter plots for genome-wide gene expressions of the evolved strains with a control or the evolved strains with cellobiose over glucose

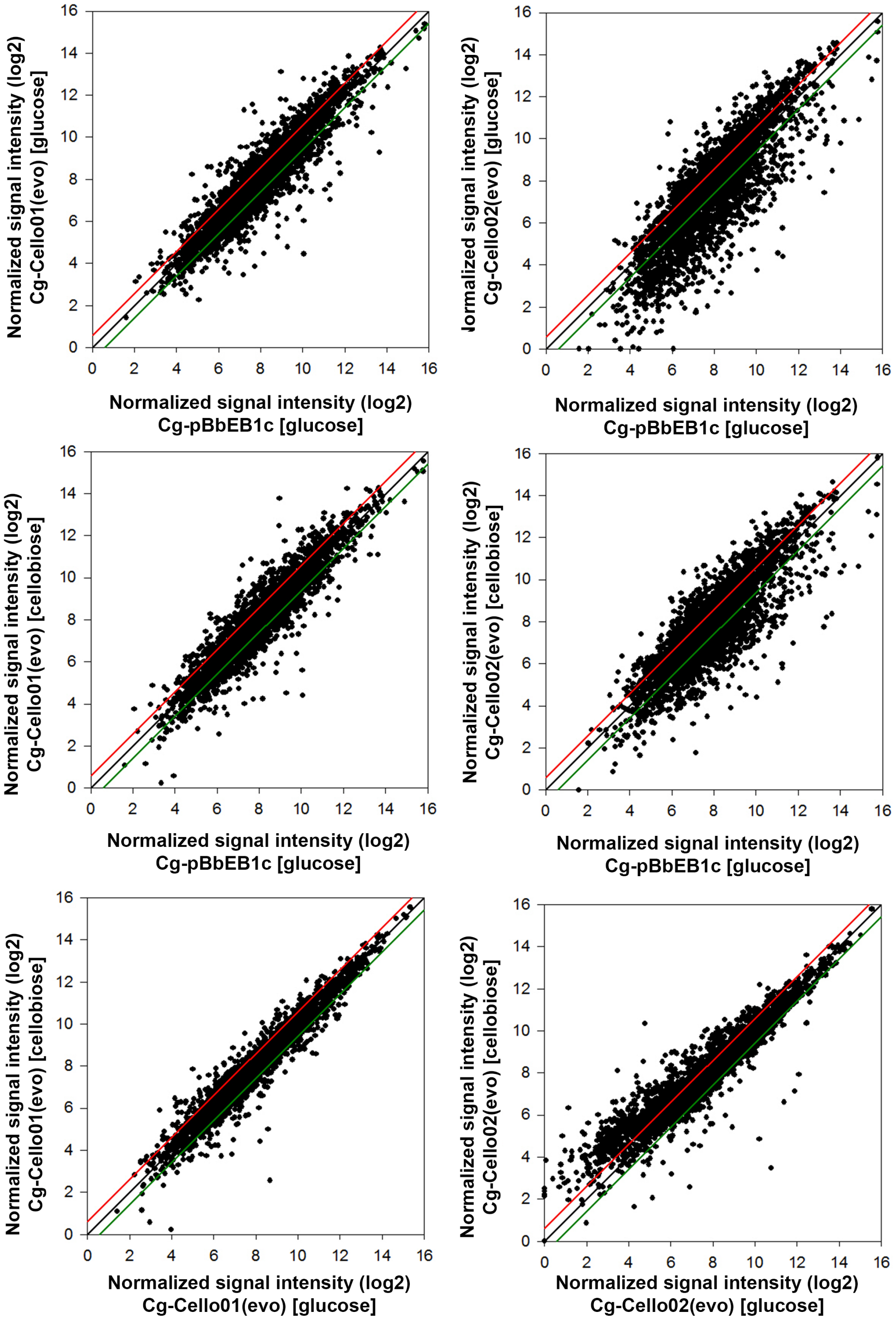

Supplement: Supplementary file 1 — 10.1186/s12934-016-0420-z Additional data and analysis for the evolved C. glutamicum strains. [file 12934_2016_420_MOESM1_ESM.pdf]
